# *Wolbachia pipientis* modulates germline stem cells and gene expression associated with ubiquitination and histone lysine trimethylation to rescue fertility defects in *Drosophila*

**DOI:** 10.1093/genetics/iyae220

**Published:** 2024-12-31

**Authors:** Catherine H Kagemann, Jaclyn E Bubnell, Gabriela M Colocho, Daniela C Arana, Charles F Aquadro

**Affiliations:** Department of Molecular Biology and Genetics, Cornell University, Ithaca, NY 14853, USA; Department of Molecular Biology and Genetics, Cornell University, Ithaca, NY 14853, USA; Department of Molecular Biology and Genetics, Cornell University, Ithaca, NY 14853, USA; Department of Molecular Biology and Genetics, Cornell University, Ithaca, NY 14853, USA; Department of Molecular Biology and Genetics, Cornell University, Ithaca, NY 14853, USA

**Keywords:** *bag of marbles*, *Wolbachia*, histone methylation, ubiquitination, oogenesis, germline stem cell

## Abstract

*Wolbachia pipientis* are maternally transmitted endosymbiotic bacteria commonly found in arthropods and nematodes. These bacteria manipulate reproduction of the host to increase their transmission using mechanisms, such as cytoplasmic incompatibility, that favor infected female offspring. The underlying mechanisms of reproductive manipulation by *W. pipientis* remain unresolved. Interestingly, *W. pipientis* infection partially rescues female fertility in flies containing hypomorphic mutations of *bag of marbles* (*bam*) in *Drosophila melanogaster*, which plays a key role in germline stem cell daughter differentiation. Using RNA-seq, we find that *W. pipientis* infection in *bam* hypomorphic females results in differential expression of many of *bam's* genetic and physical interactors and enrichment of ubiquitination and histone lysine methylation genes. We find that *W. pipientis* also rescues the fertility and germline stem cell functions of a subset of these genes when knocked down with RNAi in a wild-type *bam* genotype. Our results show that *W. pipientis* interacts with ubiquitination and histone lysine methylation genes which could be integral to the mechanism by which *W. pipientis* modulates germline stem cell gene function.

## Introduction

In female *Drosophila melanogaster*, maintaining the proper spatiotemporal expression of the *bag of marbles* (*bam*) gene product is essential for germline stem cell (GSC) cystoblast differentiation (McKearin and Spradling 1990; McKearin and Ohlstein 1995; Ting 2013). The balance of these processes results in proper maintenance of the pool of GSCs while also generating GSC daughters that enter the differentiation program to initiate oogenesis (McKearin and Spradling 1990; Ohlstein and McKearin 1997; Xie and Spradling 1998; Song *et al*. 2004). *Bam* is expressed at very low levels in GSCs, and Bam is both necessary and sufficient for GSC daughter cell differentiation (McKearin and Spradling 1990; McKearin and Ohlstein 1995; Ohlstein *et al*. 2000). Upon division, if the GSC daughter moves away from the extrinsic signaling of the stem cell niche, *bam* expression is derepressed which triggers a cascade of genetic interactions, the repression of self-renewal factors, and the cellular programming switch to differentiation. However, if *bam* is expressed in both dividing daughter cells, both will then enter the differentiation program and the germline will eventually be lost, ultimately resulting in sterility. Similarly, if *bam* is nonfunctional, the differentiation program will be blocked, GSCs will continue to self-renew and ovarioles will fill with GSC-like tumors, ultimately resulting in sterility.

A *bam* hypomorphic single amino acid coding mutant in *D. melanogaster* contains tumorous ovaries due to over proliferation of GSC-like cells, resulting in partial sterility in females (McKearin and Spradling 1990; McKearin and Ohlstein 1995; Ting 2013; Bubnell *et al*. 2021). A maternally inherited bacterial endosymbiont, *Wolbachia pipientis* infects many insects and manipulates host reproduction to increase its transmission using mechanisms such as cytoplasmic incompatibility, male killing, and feminization (Riegler *et al*. 2005; Chrostek *et al*. 2013; Truitt *et al*. 2018). Interestingly, *W. pipientis* infection of female *bam* hypomorph flies causes a partial rescue of the otherwise reduced hypomorph fertility and a reduction in overproliferating GSC-like cells (Flores *et al*. 2015; Bubnell *et al*. 2021). In contrast to females, the phenotype of male *bam* hypomorphs is fully sterile and this phenotype is not rescued by infection by *W. pipientis* (Flores *et al*. 2015). *W. pipientis* also interacts with 2 other GSC regulating genes, *Sex-lethal* (*Sxl*) (Starr and Cline 2002) and *mei-P26* (Russell *et al*. 2023), rescuing the reduced fecundity of these gene mutants. The precise mechanisms *W. pipientis* uses to rescue these GSC genes to manipulate gametogenesis remain unknown, although Russell *et al*. (2023) recently showed that the *W. pipientis* rescue of *mei-P26* loss involves restoration of proper pMad, Bam, Sxl, and Orb protein expression (Russell *et al*. 2023). Studying the interaction between *W. pipientis* and the *bam* hypomorph provides an excellent tool to understand whether *W. pipientis* employs comparable mechanisms across different contexts (e.g. the mechanisms behind the *bam* hypomorph rescue and the induction of cytoplasmic incompatibility) to manipulate reproduction.

There is abundant evidence that *W. pipientis* modulates host gene expression during infection (He *et al*. 2019; Dou *et al*. 2021; Lindsey *et al*. 2021). Therefore, it is likely that *W. pipientis* infection of *bam* hypomorphic ovaries leaves a gene expression profile of the rescue phenotype. The genotype of *W. pipientis* infecting *bam* hypomorphic females impacts the magnitude of fertility rescue: *w*MelCS-like variants exhibit higher female *bam* hypomorphic fertility rescue compared with *w*Mel-like variants (Bubnell *et al*. 2021). In males, *w*MelCS-like variants exhibit high titer compared with *w*Mel-like variants (Chrostek *et al*. 2013) and modulate host phenotypes in a titer-dependent manner. *W. pipientis* titer is known to influence a wide array of host phenotypes (Chrostek *et al*. 2013; Truitt *et al*. 2018; Bubnell *et al*. 2021) and therefore may modulate the magnitude and gene expression profile of *bam* hypomorph fertility rescue in females.

In this study, we first characterize conditions for optimal *bam* hypomorph fertility rescue by 4 *W. pipientis* variants (2 *w*MelCS with high female titer and high rescue and 2 *w*Mel with low female titer and low rescue). We next provide a comprehensive dataset and illustrate the impact of 4 distinct *w*Mel variants on gene expression in wild-type (WT) and *bam* hypomorph ovaries within an isogenic host background, contributing to the growing resources for studying the role of *W. pipientis* in *D. melanogaster* oogenesis. We identify host candidate genes and pathways involved in the *bam* hypomorph fertility rescue by *W. pipienti*s using RNA-seq to compare *W. pipientis*-infected vs uninfected *bam* hypomorph and *bam* WT genotypes. We finally functionally validate the *W. pipientis* genetic interaction with 9 of 30 candidate genes via RNAi knockdown, revealing *W. pipientis* interactions with ubiquitin and histone lysine methylation genes.

## Materials and methods

### Fly strains and husbandry

The 4 *W. pipientis* variants and uninfected control used in this study were generously provided by Luis Teixeira (Chrostek *et al*. 2013). The generation of the *bam[*L255F*]* hypomorph and our control *bam w[1118]* fly lines, which we consider as our WT line, containing each of the 4 *W. pipientis* variants, was described in the study conducted by Bubnell *et al*. (2021). Of note, the *bam* hypomorph mutation we use in our current study was recently remade using the same single amino acid change as the original *bam[BW]* hypomorph but in a *w[1118]* isogenic background (Flores *et al*. 2015; Bubnell *et al*. 2021, 2022). The female *bam* hypomorph (*w[1118]*; *bam[L255F]*/*bam[In2-3xP3DsRed]*) that was used in this study was generated by crossing the *bam[L255F]*/TM6 female to a *bam* null (*w[1118]*; *bam[In2-3xP3DsRed]*/TM6) male (Bubnell *et al*. 2021, 2022). Additionally, an uninfected *bam* hypomorph control was used along with WT *bam w[1118]* fly lines containing each *W. pipientis* variant. The *bam* null genotype we used in this study was the homozygotes from the line described above in the *w[1118]* isogenic background *w[1118]*; *bam[In2-3xP3DsRed]*/*bam[In2-3xP3DsRed]* (Bubnell *et al*. 2021).

All *D. melanogaster* lines were maintained on yeast glucose food and placed in an incubator at 25°C with a 12-h light–dark cycle.

### *W. pipientis* variants

All experiments were conducted with *D. melanogaster* lines infected with 1 of 4 *W. pipientis* variants (*w*Mel2a, *w*Mel3, *w*MelCS2a, and *w*MelCS2b) and uninfected control *D. melanogaster* lines in the WT and *bam* hypomorph genotypes. *w*Mel2a and *w*Mel3 are referred to as *w*Mel-like variants, while *w*MelCS2a and *w*MelCS2b are referred to as the *w*MelCS-like variants. PCR was used to confirm that the correct *W. pipientis* variant infected *D. melanogaster* using *W. pipientis*-specific primers listed in Riegler *et al*. (2005).

### Developmental time course and time interval fertility assay

Aged virgin WT or *bam* hypomorph females were mated with 2- to 3-day-old Canton-S males for 24 hours. We used unmated 3-day-old, mated 3-day-old, mated 6-day-old, and mated 9-day-old female WT and *bam* hypomorph females. Ten WT or *bam* hypomorph female flies and 10 Canton-S males were mated in a single vial with 9 additional replicates per age and mating status. For example, 10 2-day-old *bam* hypomorph females were mated with 10 2– to 3-day-old Canton-S males at 11 Am and collected at 11 Am the next morning.

We collected the female parents of different ages/mating statuses to measure *W. pipienti*s titer and conduct RNA-seq. Subsequently, a time interval fertility assay was conducted by measuring the number of progeny from each vial in which the parents mated. The progeny per vial were counted every 2 days for 8 days, and the sum of progeny was used for further analysis.

A Poisson response distribution was conducted in R (v. 4.1.0) at each timepoint to determine statistically significant differences in fertility between *D. melanogaster* infected with different *W. pipientis* groups (*w*Mel-like and *w*MelCS-like) and compared with the uninfected control. The equation used was as follows: Fertility (response variable) ∼ *W. pipientis* Group * Day.

### Ovary dissections and DNA/RNA extractions

Ovaries of the parent female flies from the time interval assay were dissected in cold PBS using 2 forceps. The Zymo Quick-RNA 96 kit was used to extract DNA and RNA from dissected female parent ovaries.

### *W. pipientis* titer quantification

We used DNA from parental ovaries from the time interval fertility assays for *W. pipientis* titer quantification by qPCR. We used absolute quantification, a method that compares DNA of an unknown quantity to a standard curve made from DNA of a known quantity. This method was shown to be more efficient in measuring *W. pipientis* titer than relative quantification (Christensen *et al*. 2019).

We generated a plasmid containing 5 different *W. pipientis* loci used in previous studies (Chrostek and Teixeira 2015; Flores *et al*. 2015; Shi *et al*. 2018; Costa Baião et al. 2021). We ordered a gBlock (IDT) containing the target sequences of DprA, Arm, Wsp, FusA, and Octomom (Supplementary Table 6, Supplementary Fig. 8). Each target is separated by EcoRI sites for ease of future subcloning (Supplementary Table 6, Supplementary Fig. 8). We blunt ligated the gBlock. *W. pipientis*-specific gene DNA recombination mediator protein A, DprA, was used as our target to measure *W. pipientis* titer. qPCR was run on a QuantStudio 7 Pro provided by Cornell's Biotechnology Resource Center. We used a linear regression model (R v. 4.1.0) on all of the differentially expressed genes with a *P*-value of <0.05 and an absolute log2 fold change of >1 (Supplementary File 1) at each timepoint to determine statistically significant differences in *W. pipientis* titer between *D. melanogaster* infected with different *W. pipientis* groups (*w*Mel-like and *w*MelCS-like) and between the uninfected controls. The equation we used was as follows: *W. pipientis* titer (response variable) ∼ *W. pipientis* Group * Day (R v. 4.1.0, glm.nb).

To assess correlations between *D. melanogaster* fertility and *W. pipientis* titer, we used a linear regression model. The equation we used was as follows: *D. melanogaster* Fertility ∼ *W. pipientis* Titer (R v. 4.1.0, glm.nb). Given the relationship between titer and genotype, we used a random-effect model to assess the contribution of *W. pipientis* genotype to the total variance in fertility for each *bam* genotype. The equation we used as follows: *D. melanogaster* Fertility ∼ *W. pipientis* variant (random effect) [Supplementary Table 1, lmer(Fertility ∼ (1|*W. pipientis v*ariant)), R v. 4.4.1].

### Choosing timepoints and biological replicates for RNA-seq

The time interval fertility assays showed that peak fertility rescue occurred in 6-day-old flies. Therefore, 6-day-old female flies were used for RNA-seq. Additionally, we used unmated 3-day-old female flies as a mating control and mated 3-day-old female flies as an age control for RNA-seq. We solely included an unmated 3-day-old control, as sexual maturity is in 2- to 5-day-old female *D. melanogaster*, rather than including both an unmated 3- and 6-day-old control. Four biological replicates were used for each age/mating status.

### Library preparation and 3′ RNA sequencing

Ovaries from *bam* hypomorph and WT flies infected with each *W. pipientis* variant and the uninfected controls were dissected, DNA/RNA extractions were conducted, and the RNA was subsequently used for 3′RNA-seq library construction and Illumina NextSeq500 (conducted by the Cornell Genomics Facility).

### Differential expression analysis

RNA-seq reads were mapped to the reference *D. melanogaster* genome (Flybase) using STAR (Dobin *et al*. 2013). FastQC of the FastQ files showed that quality scores were high (>30 phred score), and adapter content was low (<2% of sequence). Therefore, no reads were trimmed (Liao and Shi 2020). FastQC of the aligned sequences showed that sample alignment on average was 87%. However, 1 WT 3-day-old biological sample infected with *w*Mel3 aligned below 70% and was removed. A read count matrix was made and used for differential expression analysis using DESeq2 (Love *et al*. 2014).

WT ovaries contain a higher proportion of differentiated germ cells and developing cysts compared with *bam* hypomorph ovaries, even when rescue by *W. pipientis* occurs. Considering that there are significant differences in the cell types and developmental stages between the *bam* hypomorph and WT ovaries, we did not call differential expression between these genotypes directly. Therefore, differential expression analyses compared *W. pipientis*-infected *D. melanogaster* expression relative to uninfected *D. melanogaster* expression within *bam* genotypes. We then compared the genes we identified as differentially expressed within *bam* genotypes between the WT and *bam* hypomorph genotypes.

## Protein–protein networks

STRING (Version 12.0) was used to identify the protein–protein interactions between the differentially expressed genes in the *bam* hypomorph and WT genotypes (Figs. 3 and 4, Supplementary Figs. 2 and 5). Subsequently, we used Cytoscape (version 3.9.1) to create the protein–protein network (Figs. 3 and 4, Supplementary Figs. 2 and 5).

### Ovarian tissue composition, mating, and age analyses

We identified genes whose expression was influenced by the higher proportion of differentiating and developing egg chambers that results from *W. pipientis* rescue of oogenesis computationally using glm.nb. Specifically, a null formula containing expression as the dependent variable and infection status as an independent variable was compared with a test formula containing expression as the dependent variable and infection status and oogenesis rescue as independent variables. We used a ranking for *W. pipientis* rescue in the *bam* hypomorph we determined from the time interval fertility assay to represent the increase in the proportion of differentiating and developing egg chambers as the rescue of the fertility phenotype increases.

Glm.nb, a function in the MASS R package, was used to identify genes impacted by age and mating. The null formula included expression as the dependent variable and the *W. pipientis* variant as the independent variable. The test formula included expression as the dependent variable along with *W. pipientis* variant and age/mating as the independent variables. Genes impacted by age, mating, or ovarian tissue composition were then identified and are noted in Supplementary Table 2. However, we did not remove the genes from the age, mating, and ovarian tissue composition analysis from our dataset as it is possible that these genes could be differentially expressed as a consequence of age, mating, or tissue composition in addition to contributing the *bam* hypomorph fertility rescue by *W. pipientis*.

### Gene Ontology analyses

Gene ontology (GO) analyses of all differentially expressed genes from Supplementary Figs. 4 and 5 were conducted using gseGO within the clusterProfiler package in R (v.1.4. 1717). Differentially expressed genes with a *P*-value > 0.05 were removed prior to the analysis. The genes were then ranked by log2 fold change prior to using gseGO. Our gseGO analysis included all gene ontologies (biological process, cellular component, and molecular function).

### RT-qPCR of candidate genes from RNA-seq analysis in *bam* null ovaries

We dissected ovaries from 10 3-day-old females per biological replicate in ice-cold 1 × PBS. The ovaries were immediately placed in NEB DNA/RNA stabilization buffer and then stored at −80°C until RNA extraction. We used the NEB total RNA mini-prep kit according to the manufacturer's protocol and included the on-column DNase treatment.

We used the Luna One Step RT-qPCR mastermix from NEB according to the manufacturer's instructions at 10 μL total volume in 384 well plates. The primers we used for each gene are listed in Supplementary Table 6. We used the QuantStudio 7 Pro qPCR machine with the appropriate cycle settings as described in the Luna One Step RT-qPCR kit. We included a standard curve for each target on every plate at a 1:5 dilution and then used the standard curve to correct for different primer efficiencies. We used *Rpl32* as our housekeeping control gene for each target gene and calculated the relative quantity of each gene comparing uninfected to *W. pipientis-*infected samples using the delta–delta CT method (Ho *et al*. 2019). We determined statistical significance of the mean difference in expression between infected and uninfected samples using estimation statistics with Dabest (Ho *et al*. 2019) and bootstrap resampling (5,000 resamples) with a 95% confidence interval (CI) cutoff.

### Gal4 drivers and UAS-RNAi lines of the candidate genes

*W. pipientis* were introduced into the Gal4 drivers by crossing the second chromosome GAL4 driver (BDSC line: 4442) with female *w[1118]*; TM2/TM6 flies infected with *w*Mel59 or the third chromosome GAL4 driver (BDSC line: 4937) with female *w[1118]* flies infected with *w*MelCS2b (Supplementary Table 5). The UAS and Gal4 driver genotypes and BDSC stock numbers are listed in Supplementary Table 5.

### Immunostaining of GAL4/UAS knockdowns

To immunostain the Gal4–UAS candidate gene flies (*W. pipientis* infected and uninfected), we aged the flies for 2–5 days, mated them with WT Canton-S males for 1–2 days (within the 2–5 days as done in our time interval fertility assays), and dissected their ovaries. We used the protocol used in Wenzel and Aquadro (2023) for immunostaining; however, we used 0.2% Triton-X 100 in the PBTA. We used anti-Vasa [antirat, 1:20, Developmental Studies Hybridoma Bank (DSHB)] and anti-Hts-1B1 (antimouse, 1:40, DSHB). We used the following secondary antibodies: Alexa488 (goat antirat, Invitrogen cat no.: A-11006) and Alexa568 (goat antimouse, Invitrogen cat no.: A-1103) all at 1:500. A Zeiss i880 confocal microscope was used for all imaging with the 488- and 568-nm laser lines at 10 × or 40 × (Plan-Apochromat 1.4 NA, oil) (Cornell BRC Imaging Core Facility).

### Fertility assays of GAL4/UAS knockdowns

Virgin females of each genotype listed in Supplementary Table 4 and virgin WT Canton-S males were collected and aged for 2–5 days. 30–50 crosses of 1 female and 2 males were conducted using vials containing yeast-glucose food. The vials were placed in a 25°C incubator with a 12 hour light/dark cycle. Progeny were counted every other day over 7–8 days, starting on the day of first eclosion, and the parents were subtracted from the counts. Estimation statistics, a nonparametric resampling-based statistical test, were used to determine the mean difference of the number of progeny (effect size) between *W. pipientis*-infected vs uninfected females or between the *W. pipientis* uninfected test and control genotype. Significance was stated if the mean difference was outside of the 95% bootstrap (with 5,000 resamples) CI. Estimation statistics were conducted using Dabest version Ondeh (v2024.03.29) in Python with 5,000 bootstrap resamples (Ho *et al*. 2019).

## Results

### Assessment of the effect of *W. pipientis* variant and *D. melanogaster* female age after mating on *W. pipientis* rescue of the *bam* hypomorph fertility defect

**Fig. 1.**
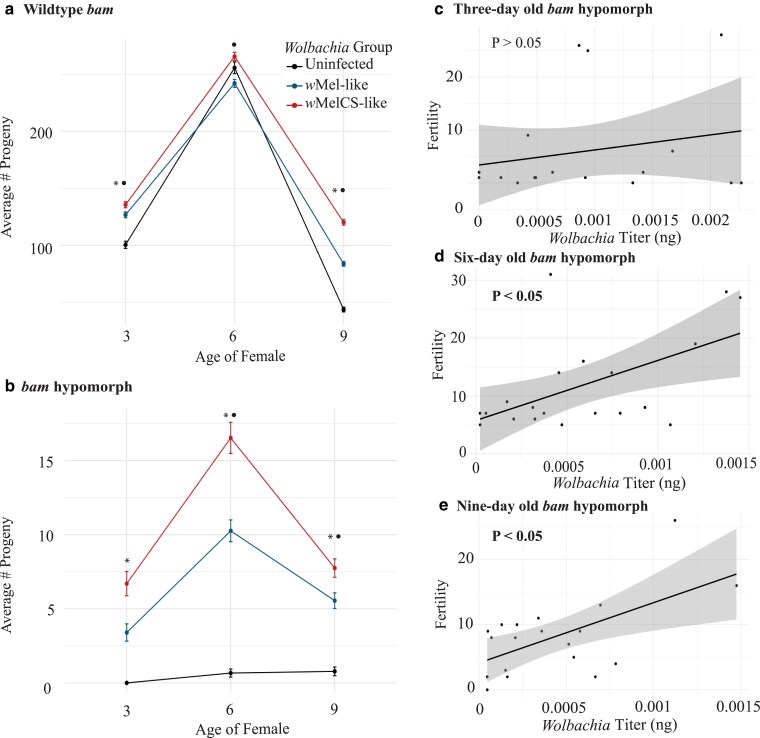
Fertility in the *bam* hypomorph flies shows that fertility rescue is dependent on the age of the female fly and *W. pipientis* genotype and that titer is positively correlated with fertility in 6- and 9-day-old flies. The estimated marginal mean number of progeny per *W. pipientis* group (*w*Mel-like: *w*Mel2a and *w*Mel3, *w*MelCS-like: *w*MelCS2a and *w*MelCS2b) infecting *D. melanogaster* and the uninfected *D. melanogaster* control in the a) *bam* WT and b) *bam* hypomorph genotypes. The *x*-axis shows the age of the female fly after a day of mating, the *y*-axis (note the difference in *y*-axes between a and b) shows the mean number of progeny, and the bars represent the SE of each *W. pipientis* group at a given age. The symbol “*” denotes a statistically significant difference between the uninfected control and the *W. pipientis*-infected samples at each given age. The symbol “●” denotes a statistically significant difference between *W. pipientis* groups (*w*Mel-like vs *w*MelCS-like) at each given age. c)–e) *Bam* hypomorph fertility as a response to the combined *W. pipientis* titer of all 4 *W. pipientis* variants infecting *D. melanogaster*. Fertility and combined titer across *W. pipientis* infecting mated aged flies: c) 3-day-old, d) 6-day-old, and d) 9-day-old females (linear regression model Fertility ∼ Titer, *P* > 0.05). The *P*-value indicates whether fertility and titer are statistically correlated.

We first asked whether infection with putative low- and high-titer *W. pipientis* variants was associated with increased fertility in WT and *bam* hypomorphic females at 3 different aged timepoints (Fig. 1a and b). While our focus is on the *bam* hypomorph, the WT *bam* genotype was used as a baseline for the effect of age on titer and fertility. We measured the fertility of 3-, 6-, and 9-day-old WT uninfected *D. melanogaster* as well as those infected with either of 2 putative low-titer *w*Mel*-*like variants (*w*Mel2a and *w*Mel3) and either of 2 putative high-titer *w*MelCS-like variants (*w*MelCS2a and *w*MelCS2b) by counting the number of adult progeny per 10 females (Fig. 1a).

We observed the highest number of adult progeny in 6-day-old WT *bam* females, which we refer to as “peak fertility” (Fig. 1a and Supplementary Table 1). However, *W. pipientis* infection did not increase fertility in this age group (Fig. 1a, Poisson’s response distribution, *P* > 0.05). *W. pipientis* infection in 3- and 9-day-old WT *bam* females led to a statistically significant increase in fertility across all variants (Fig. 1a, Poisson’s response distribution, *P* < 0.05). The putative high-titer *w*MelCS-like variants were associated with higher fertility than the putative low-titer *w*Mel-like variants at all ages (Fig. 1a, Poisson’s response distribution, *P* < 0.05).

In *bam* hypomorph females, we also observed peak fertility at 6 days old, but fertility never reached *bam* WT levels (Fig. 1b and Supplementary Table 1). In 6- and 9-day-old *bam* hypomorph females, *W. pipientis* infection across all variants significantly increased fertility (Fig. 1b, Poisson’s response distribution, *P* < 0.05). In contrast, *W. pipientis* infection did not increase fertility of 3-day-old females (Fig. 1b, Poisson’s response distribution, *P* > 0.05). Like the *bam* WT genotype, the putative high-titer *w*MelCS-like variants were associated with higher fertility than the putative low-titer *w*Mel-like variants at all ages (Fig. 1b, Poisson’s response distribution, *P* < 0.05).

Next, we measured *W. pipientis* titer in addition to fertility for both WT *bam* and *bam* hypomorph females individually infected with the putative low-titer *w*Mel-like (*w*Mel2a and *w*Mel3) variants and the putative high-titer *w*MelCS-like (*w*MelCS2a and *w*MelCS2b) variants. We used DNA from parental ovaries from the time interval fertility assays explained above for *W. pipientis* titer quantification by qPCR. We used a linear regression model in mated 3-, 6-, and 9-day-old flies to test the effect of *W. pipientis* titer (combined across all 4 variants) on fertility. For *bam* WT females, while we observed a positive relationship between female fertility and ovarian *W. pipientis* titer across all ages, none of these correlations were statistically significant (Supplementary Fig. 1, linear regression model Fertility ∼ Titer, *P* > 0.05).

In contrast to *bam* WT females, for *bam* hypomorph females, we observed a significant positive correlation between female fertility and ovarian *W. pipientis* titer in 6- and 9-day-old females as well as a positive (but not statistically significant) relationship in 3-day-old females (Fertility ∼ Titer, Fig. 1c–e).

Given the relationship between titer and genotype, we used a random-effect model to assess the contribution of *W. pipientis* genotype to the total variance in fertility for each *bam* genotype [Fertility ∼ (1|*W. pipientis v*ariant), Supplementary Table 1]. *W. pipientis* genotype contributed very little to the variance in fertility for WT *bam* females (intercept at 0), and for *bam* hypomorphic females, 7.5% of the variance in fertility is due to *W. pipientis* genotype. Therefore, the effect of titer on fertility for both *bam* genotypes is not likely due to the effect of *W. pipientis* genotype alone. High- and low-titer *W. pipientis* variants are thus representative of the effects of high and low *bam* hypomorph fertility rescue (Bubnell *et al*. 2021).

### GSC regulating genes are not differentially expressed in *bam* hypomorphic females as a result of mating status or differences in ovarian tissue developmental stages

To define the relationship more reliably between *W. pipientis* titer, female fertility, and gene expression, we took these measurements from the same female flies, thereby reducing technical variability. To do this, we prepared mRNA from ovaries of unmated 3-day-old, mated 3-day-old, and 6-day-old flies infected with each *W. pipientis* variant (*w*Mel2a*, w*Mel3*, w*MelCS2a*, w*MelCS2b) in both *bam* genotypes (WT and hypomorph) and performed 3′ RNA-seq. Given this design, and to reduce including false-positive candidate loci for the *W. pipientis bam* hypomorph rescue gene expression profile, we first asked whether the variables of age, mating status, or differences in ovarian tissue developmental stages were associated with differential expression of GSC regulating genes.

We included unmated 3-day-old flies in our RNA-seq analysis to computationally identify genes that were differentially expressed due to mating rather than the *W. pipientis* fertility rescue of *bam* hypomorphic females. To balance the cost of additional samples and generating informative data, we chose to include this single age point, which we predict to be most informative on the effect of mating, as the age of sexual maturity is 2–5 days old for female *D. melanogaster* (Markow and O’Grady 2006). We additionally used the 6-day-old timepoint for *bam* hypomorph and WT females to determine what genes were impacted by the age of the fly.

Between *w*Mel and *w*MelCS-like infected *bam* hypomorph ovaries, there are subtle differences in the development and morphology of the tissue due to greater rescue of the overproliferating GSC mutant phenotype by *w*MelCS compared with *w*Mel. Consequently, the greater the rescue of the *bam* hypomorph phenotype by *W. pipientis*, the greater the proportion of properly developing egg chambers compared with GSC-like tumors in the ovary. Therefore, we computationally predicted genes differentially expressed due to differences in tissue development, female age, and mating status to better filter for genes whose expression was impacted by infection with *W. pipientis*. We fit our data to a negative binomial generalized linear model for both analyses.

After multiple hypothesis testing, we identified 35 genes that were differentially expressed in the *bam* hypomorph and 17 in the WT genotype due to age and/or mating or differences in ovarian tissue composition (Supplementary Table 2). Of these genes in either genotype, none were *bam's* genetic or physical interactors or one of the 366 GSC-related genes identified by Yan *et al*. (2014).

### Analysis of all differentially expressed genes associated with *W. pipientis* infection in *bam* WT and *bam* hypomorph females

**Fig. 2.**
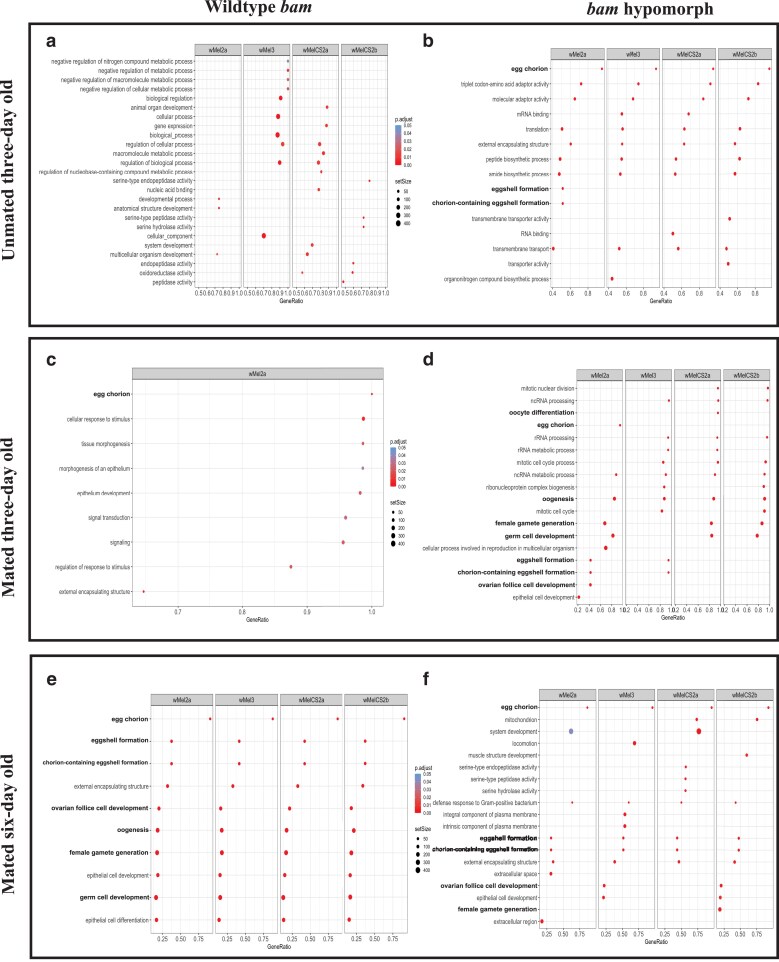
Gene enrichment analysis of all host genes from RNA-seq of WT and *bam* hypomorph ovaries by age of female flies (*P* < 0.05, abs log2 fold change > 1). The top 10–15 GO terms from a GO enrichment analysis including all differentially expressed (*D. melanogaster* infected with each *W. pipientis* variant compared with uninfected *D. melanogaster*) host genes from a) WT unmated 3-day-old flies, b) *bam* hypomorph unmated 3-day-old flies, c) WT mated 3-day-old flies, d) *bam* hypomorph mated 3-day-old flies, e) WT mated 6-day-old flies, and f) *bam* hypomorph mated 6-day-old flies (gseGO in R). Each column represents the *W. pipientis* variant infecting *D. melanogaster*. The set size represents the number of genes within each GO category. The gene ratio is the number of genes within the GO term (set size) divided by the total number of differentially expressed genes. GO categories related to reproduction are highlighted in bold.

Next, we used this RNA-seq dataset to ask whether *W. pipientis* altered the gene expression of any host genes in ovaries of the female flies whose fertility and *W. pipientis* titer we measured above. We first conducted a gene enrichment analysis of all host genes (Fig. 2). We generated gene enrichment profiles for each age, mating status, and *bam* genotype infected with each *W. pipientis* variant using clusterProfiler (Wu *et al*. 2021). The age in which we observed the greatest enrichment of genes in reproduction-related GO categories differed between the 2 *bam* genotypes: mated 3-day-old flies for the *bam* hypomorphic genotype (Fig. 2d) and mated 6-day-old flies for the *bam* WT genotype (Fig. 2e). Of particular interest to us was that *bam* hypomorph infection by *W. pipientis* variants was associated with enrichment of over 100 genes involved in oogenesis, germ cell development, female gamete generation, and other oocyte-specific GO terms in mated 3-day-old flies (Fig. 2d). While enrichment of genes involved in follicle cell development and female gamete generation occurred in 6-day-old *bam* hypomorph flies, they were only enriched in *w*Mel3- and *w*MelCS2b-infected flies (Fig. 2f). Genes enriched in unmated 3-day-old *bam* hypomorph flies include egg chorion-related genes, genes involved in RNA processes, and other biosynthetic processes (Fig. 2b).

**Fig. 3.**
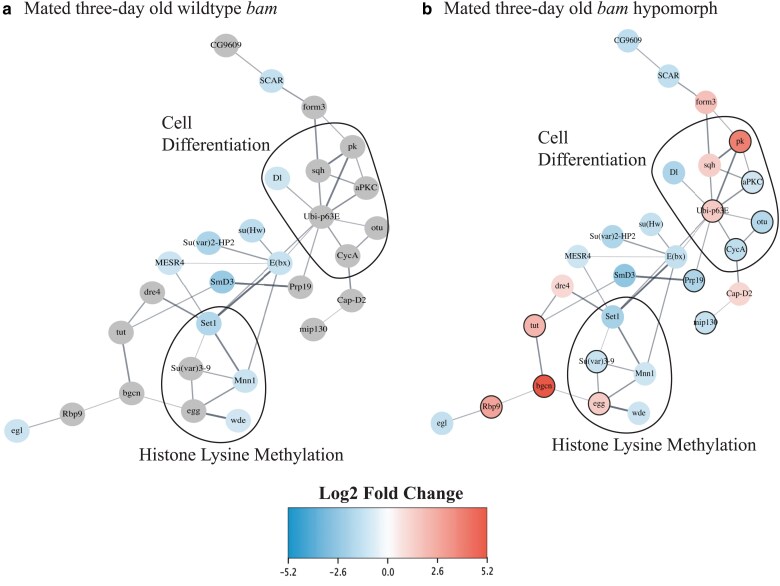
Differential expression of GSC genes shows enrichment of genes involved in cell differentiation and histone lysine methylation in the *bam* hypomorph. The differentially expressed (*D. melanogaster* infected with each *W. pipientis* variant compared with uninfected *D. melanogaster*) GSC genes identified from Yan *et al*. (2014) in a) mated 3-day-old WT and b) *bam* hypomorph flies plotted in a protein interaction network consisting of the genes that were differentially expressed in the *bam* hypomorph (Cytoscape). The same protein–protein network was used for the WT genotype for comparison. The average log2 fold change of each differentially expressed gene between flies infected with each *W. pipientis* variant was used as there was not a significant difference between differential expression of these genes in *Drosophila* infected with *w*Mel-like and *w*MelCS-like variants. Genes that are only differentially expressed in the *bam* hypomorph are circled with a black border. The top GO categories as identified from Cytoscape's enrichment analysis were circled within each network.

The *w*Mel-like and *w*MelCS-like variants of *W. pipientis* exhibit distinct phenotypic effects in *D. melanogaster*, encompassing variation in viral protection, fecundity, and cytoplasmic incompatibility. We hypothesized that *W. pipientis* variant-specific infected gene enrichment could reveal processes that explain the difference in fertility rescue between *w*Mel-like vs *w*MelCS-like infected *bam* hypomorph females. Our initial differential expression analyses were between *D. melanogaster* infected with each *W. pipientis* variant compared with the uninfected *D. melanogaster* control. Next, to compare *W. pipientis* groups to one another, we combined the differentially expressed genes from each *W. pipientis* group for analysis. Gene enrichment analysis of oogenesis-related genes in 3-day-old *bam* hypomorph flies, prior to peak fertility rescue, revealed variation between flies infected with different *W. pipientis* variants (Supplementary Fig. 3). Unique to ovaries infected with *w*Mel-like *W. pipientis* variants, we observed downregulation of genes involved in egg formation and oogenesis-related GO categories (Supplementary Fig. 3b, Supplementary Table 2). We did not observe this pattern for any of the other age/mating status comparisons or in the WT *bam* background (Fig. 3a and c, Supplementary Fig. 4, Supplementary Table 3).

### Analysis of 366 functionally validated GSC genes associated with *W. pipientis* infection in *bam* WT and *bam* hypomorph females

To focus our analysis on genes most likely to interact with *bam's* function in GSC daughter cell differentiation, we next used a dataset of 366 genes that have functionally defined roles in GSC differentiation, maintenance, and other processes in oogenesis plus *bam's* additional 25 known genetic and physical interaction partners identified in Flybase (Yan *et al*. 2014). The 366 genes include 11 of Bam's 36 genetic and physical interactors identified from Flybase (Yan *et al*. 2014; Thurmond *et al*. 2019). A gene interaction network of all 391 genes is shown in Supplementary Fig. 2 (Cytoscape). We investigated whether these genes were differentially expressed in our WT *bam* and *bam* hypomorph RNA-seq datasets by comparing each *W. pipientis* variant-infected *bam* genotype to uninfected flies of the same *bam* genotype. Using these within *bam* genotype differential expression calls, we additionally examined how gene expression varied across *bam* genotypes, *W. pipientis* infection status, and age. Finally, we constructed gene networks of all the differentially expressed genes among the subset of 391 genes (Supplementary Fig. 5). We found differentially expressed genes between infected and uninfected ovaries across all ages and within the *bam* hypomorphic and WT *bam* genotypes [Benjamini and Hochberg (BH) adjusted *P*-value <0.05, log2 fold change ≥ 1, Supplementary Table 4, Supplementary Fig. 5, Supplementary File 1].

Of the differentially expressed genes specific to the *bam* hypomorph, enrichment of GSC daughter cell differentiation genes solely occurred in mated 3-day-old flies (Fig. 3, Supplementary Fig. 5), prior to the age we observed peak fertility rescue (Fig. 1b). This is consistent with the timing of the development of a GSC daughter cell to a fully differentiated oocyte (∼66 h) (Lin and Spradling 1993), and thus, we expected a lag between an increase in fertility caused by a gene expressed in GSCs and early cystoblasts. Of the *bam* hypomorph-specific differentially expressed genes in mated 3-day-old flies (Fig. 3b), 7 genes (*Ubi-p63E, CycA, otu, aPKC, sqh, Dl,* and *pk*) are classified in the cell differentiation GO category and 5 genes (*Ubi-p63E, CycA, otu, Dl,* and *pk*) are also involved in ubiquitin-related processes. Two genes are classified in the histone lysine methylation category (*egg* and *su(var)3-9*), a process necessary for GSC daughter cell differentiation and oogenesis (Fig. 3b) (Yoon *et al*. 2008; Koch *et al*. 2009; Wang *et al*. 2011; Smolko *et al*. 2018).

### Analyzing Bam's 36 genetic and physical interaction partners alone highlights differential expression of ubiquitin-associated genes

*Bam* physically binds to its protein partners and functions as a complex to promote differentiation and repress self-renewal (Li *et al*. 2013). Bam can also directly bind ubiquitin without a partner (Cai *et al*. 2023). There are extensive data on genetic and physical interactions between all *D. melanogaster* genes. We thus next asked which of Bam's 36 documented physical and genetic interaction partners (Thurmond *et al*. 2019) are differentially expressed between uninfected and infected *bam* hypomorph ovaries [11 of the 36 genes were also identified in the Yan *et al*. (2014) RNAi screen]. If *bam* or any of Bam's documented physical and genetic interaction partners are differentially expressed, they are candidates for the mechanism of the *bam–W. pipientis* interaction.

**Fig. 4.**
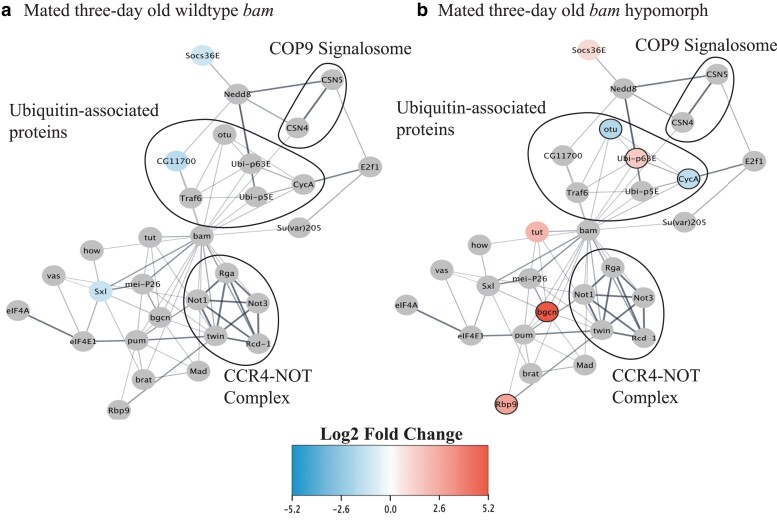
Differential expression of solely Bam's physical interactors shows enrichment of genes involved in ubiquitination. Bam's differentially expressed (*D. melanogaster* infected with each *W. pipientis* variant compared with uninfected *D. melanogaster*) physical interactors in the a) mated 3-day-old WT and b) *bam* hypomorph genotypes. Three of the largest protein complexes known to be associated with Bam are circled: COP9 signalosome, ubiquitin-associated proteins, and the CCR4-NOT complex. Bam's interactors that are not differentially expressed are annotated with gray circles.

We generated a separate gene network of only *bam's* known physical interaction partners and found that 3 out of 6 differentially expressed genes were involved in ubiquitination or deubiquitination in unmated 3-day-old *bam* hypomorph females but not *bam* WT females (Fig. 4). Given our finding that *W. pipientis* infection was associated with the differential expression of ubiquitin- and histone lysine trimethylation-associated genes (as seen from the 391 gene dataset analysis), we next investigated whether *bam's* differentially expressed interaction partners have documented roles in Bam's ubiquitin binding function, they bind ubiquitin on their own, or they have any evidence of binding histones or other chromatin modifications.

**Fig. 5.**
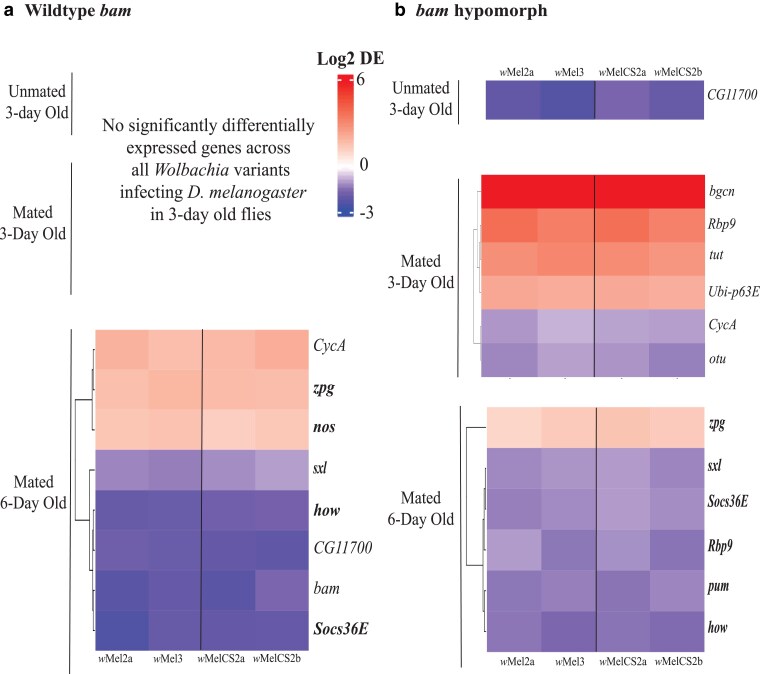
*W. pipientis* infection causes differential expression of *bam's* genetic and physical interactors in the *bam* hypomorph. Differential expression of *bam* and *bam's* genetic/physical interactors in *W. pipientis*-infected *D. melanogaster* compared with uninfected *D. melanogaster* in the a) *bam* WT and b) *bam* hypomorph genotypes at different age/mating statuses (*P* < 0.05, abs log2 fold change >1). Each column represents the *W. pipientis* variant infecting the *D. melanogaster* shown at the bottom with a line separating the *w*Mel-like and *w*MelCS-like variants. Genes in bold are differentially expressed in both genotypes. Only genes differentially expressed across all *W. pipientis* infecting the WT or *bam* hypomorph genotype are shown (refer to Supplementary File 1 for all differentially expressed genes).

In the *bam* hypomorph genetic background, we observed differential expression for some, but not all, of *bam*'s known genetic and physical interaction partners between the *W. pipientis*-infected and uninfected females in unmated 3-, mated 3-, and mated 6-day-old flies (inferred using DESeq2, *P* < 0.05, absolute log2 fold change >1, Fig. 5). Five of the differentially expressed genes (*CycA, otu, Ubi-p63E, CG11700,* and *Socs36E*) are involved in *bam's* ubiquitin functions and 3 (*pum, Sxl,* and *zpg*) are associated with histone lysine trimethylation.

In contrast, in the *bam* WT genotype, we only observed *W. pipientis-*associated differential expression of *bam* and *bam's* genetic and physical interactors in mated 6-day-old flies (Fig. 5 and Supplementary Table 4, inferred using DESeq2, *P* < 0.05, absolute log2 fold change >1). Of the differentially expressed genes, 4 (*CycA, otu, CG11700,* and *Socs36E*) were ubiquitin-associated genes and 2 (*pum* and *sxl*) were histone lysine methylation-associated genes. The ubiquitin- and histone lysine methylation-associated genes that were differentially expressed in 6-day-old WT flies were also differentially expressed in 3-day-old *bam* hypomorph flies, suggesting the importance of these regulatory mechanisms in oogenesis across genotypes.

### *W. pipientis* infection also results in differential expression of 6 of 9 candidate GSC regulating genes independent of *bam* function

**Fig. 6.**
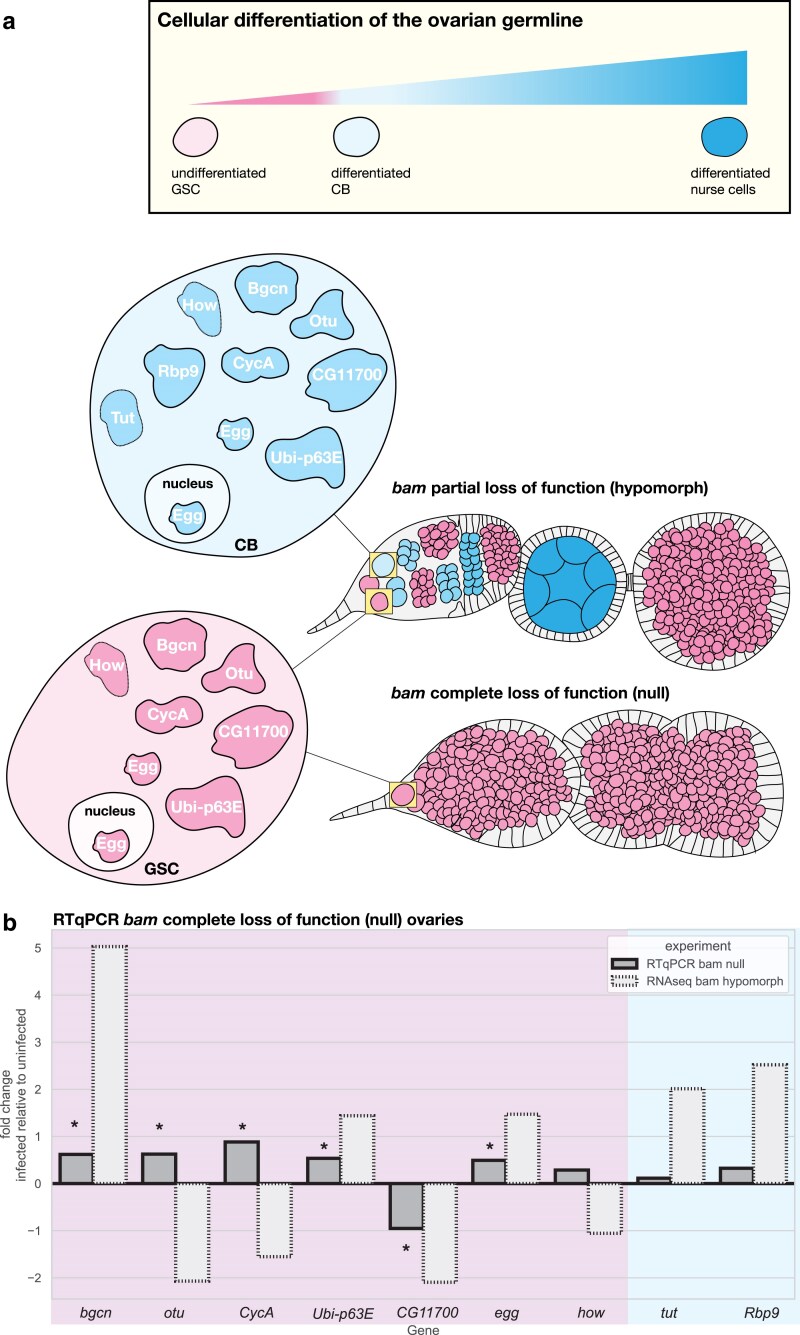
*W. pipientis* infection results in the differential expression of a subset of RNA-seq candidate genes independent of *bam* rescue. a) A schematic of the germarium and early-stage egg chambers of *D. melanogaster* ovarioles illustrating the cellular and morphological differences between *bam* hypomorphic and *bam* null tissues. The undifferentiated GSCs and early differentiated cystoblast (CB) daughter cells are located at the anterior tip of the germarium in *bam* hypomorphic females; however, as *bam* is necessary for GSC daughter cell differentiation, *bam* null ovaries contain only undifferentiated GSC-like cells and thus are fully sterile. The candidate genes from the RNA-seq analysis are shown in the cell types in which they are expressed. Only *Rbp9* and *tut* are not expressed in GSCs in addition to CBs. Egg localizes to the nucleus as well as the cytoplasm. b) Differential expression of candidate genes involved in the rescue of *bam* by *W. pipientis* between infected (*w*MelCS2b) and uninfected *bam* null ovaries using RT-qPCR with the RNA-seq log2 fold change for the same gene from the RNA-seq *bam* hypomorph analysis plotted alongside for comparison. Fold change > 0 indicates *W. pipientis* infection is associated with increased expression of that transcript, and fold change < 0 indicates *W. pipientis* infection results in decreased expression. The RT-qPCR analysis results are shaded dark gray with solid bars, and the RNA-seq analysis results are light gray with dotted lines. Colored shading behind the bars maps to the cell types (pink: GSC and CB or blue: CB only) in which those genes are expressed as outlined in the schematic above. All genes here were statistically significant in the RNA-seq *bam* hypomorph analysis. Stars indicate genes that are statistically significant in the RT-qPCR *bam* null analysis [Delta-delta cycle threshold (DDCT), 95% CI permutation test].

By design, our RNA-seq study captured genes that *W. pipientis* manipulates to rescue *bam* hypomorph fertility in addition to genes that are affected by fertility rescue and repression of the GSC-like cell overproliferation phenotype. While *bam* hypomorph ovaries are a mix of mutant GSC-like overproliferating cells and WT-like differentiating cells (i.e. cystoblasts, nurse cells, oocyte), the ovaries of *bam* null females both infected and uninfected with *W. pipientis* consist of only GSC-like cells. As *W. pipientis* infection does not simply bypass the requirement for Bam to initiate cystoblast differentiation, without Bam, GSC daughter differentiation is blocked and cannot be rescued (Fig. 6a). Therefore, candidate genes that are also differentially expressed due to *W. pipientis* infection in a complete *bam* loss-of-function background are more likely to be genes that contribute to the *W. pipientis* rescue of *bam's* differentiation function, not genes that are differentially expressed as a consequence of rescue.

Given results of both this paper and previous literature demonstrating *W. pipientis*' influence on GSC differentiation, ubiquitin-related processes, and chromatin modification (Lu *et al*. 2013; LePage *et al*. 2014; Flores *et al*. 2015; Beckmann *et al*. 2017; Lindsey *et al*. 2018; Bhattacharya *et al*. 2021; Bubnell *et al*. 2021; Kaur *et al*. 2022; Zong *et al*. 2022), we chose a subset of 9 (*bgcn, otu, CycA, Ubi-p63E, tut, egg, CG11700, how,* and *Rbp9*) out of the 30 candidate genes we identified above. For each gene, we used RT-qPCR to measure the relative quantity between *w*MelCS2b-infected and uninfected *bam* null ovaries in the same isogenic background that we used for the RNA-seq analysis (Fig. 6b and Supplementary Fig. 6). We found that *bgcn, otu, CycA, Ubi-p63E, egg,* and *CG11700* were all also differentially expressed in the *bam* null ovaries, but *tut, how,* and *Rbp9* were not significantly differentially expressed (Fig. 6bB, Supplementary Fig. 6, 95% CI bootstrap resampling). Therefore, *W. pipientis* modulates *bgcn, otu, CycA, Ubi-p63E, egg,* and *CG11700* expression even in the absence of *bam* rescue and subsequent GSC differentiation, while the effect of *W. pipientis* on *tut, how,* and *Rbp9* is each dependent on *bam* function.

These results together indicate that *W. pipientis* infection is associated with significant differential expression of ubiquitin-associated genes (*otu, CycA, Ubi-p63E,* and *CG11700*), as well as a gene likely necessary for *bam's* ubiquitin-associated functions (*bgcn*), and a histone lysine trimethylation gene (*egg*) independent of *bam* function. These genes are thus likely differentially expressed due to *W. pipientis* infection rather than the downstream effect of *W. pipientis* on GSC daughter cell differentiation and fertility rescue. Therefore, this gene set contains good candidates for the mechanism of *W. pipientis*' rescue of *bam* hypomorph fertility.

### *W. pipientis* rescues RNAi-induced GSC mutant phenotypes of several candidate genes linked to ubiquitin, histone lysine trimethylation, and GSC maintenance

We next used RNAi to knockdown 9 genes we identified as candidates in our GSC and early cystoblast analyses in a WT *bam* background. We further chose to also knockdown *how*, a gene that displayed differential expression in both *bam* genotypes during peak fertility rescue (Fig. 5), to assess whether a gene differentially expressed in a functional *bam* background also might interact with *W. pipientis*.

**Fig. 7.**
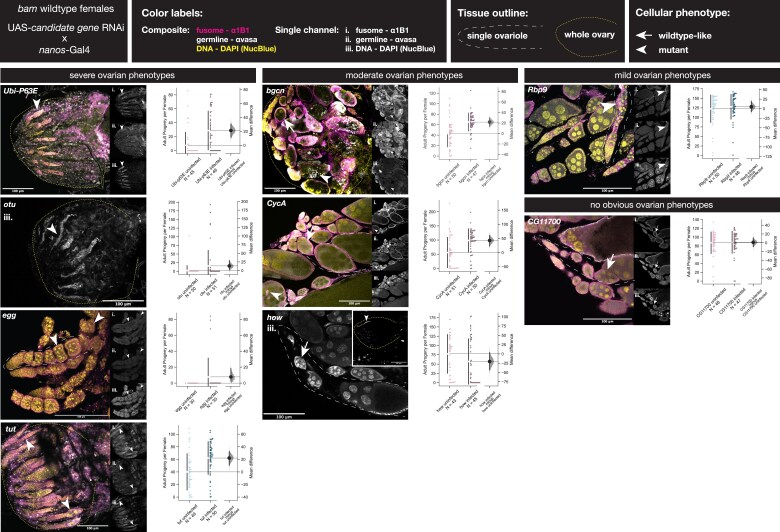
RNAi knockdown of candidate genes in WT *bam* females induces a range of ovarian phenotypes and reduced fertility, a subset of which are rescued by *W. pipientis* infection. Ovarian cytology and female fertility of candidate gene knockdowns in GSCs and early cystoblasts. Ovarian tissue is from the *nanos-*GAL4×UAS candidate gene RNAi genotype listed for each image. Ovarian defects for each knockdown are categorized as severe, moderate, mild, or no defect. Composite images are from immunostaining with antibodies to hts-1B1 (spectrosome/fusome, magenta), vasa (germline, white), and DAPI (NucBlue). Single-channel images are shown in grayscale—i. hts-1B1, ii. vasa, and iii. DAPI (NucBlue). Yellow dotted lines outline the entire ovary, and white dashed lines outline single ovarioles. Arrows point to WT cellular phenotypes, and arrowheads point to mutant cellular phenotypes. Scale bars are 100 μm. Fertility assay data for each gene knockdown are beside each corresponding ovary image. Fertility is reported as the mean difference of adult progeny per female between uninfected and infected female lines. Significance is reported as a mean difference > 0 outside of the 95% CI obtained by a bootstrap resampling test (5,000 resamples). The color of the swarm plot points corresponds to whether that gene is expressed in GSCs and CBs (pink, Fig. 5) or only CBs (blue, Fig. 5). The specific genotypes of each knockdown and their given *nanos*-Gal4 control are given in Supplementary Table 6.

We first determined the conditions necessary to generate a hypomorph-like phenotype of each gene knockdown in GSCs and cystoblasts using 2 different *nanos*-Gal4 drivers at varying temperatures to drive UAS-RNAi lines of each gene (Supplementary Table 5). We did not observe any cytological phenotype for *CG11700* knockdown females (Table 1, Fig. 7 arrows). As no mutant phenotype has been described in ovaries for *CG11700*, we checked where *CG11700* has been reported to be expressed and found that the RNAi targeted an isoform that is highly expressed in testes, with low expression in ovaries of WT *D. melanogaster* (Flybase FB2024_02, Supplementary Fig. 7). Therefore, it is likely we did not knockdown the female-enriched *CG11700* transcript, but we included the genotype in our full analysis and report the findings below.

**Table 1.** RNAi knockdowns of candidate genes reveals *W. pipientis* rescues GSC mutant phenotypes linked to ubiquitin, histone lysine methylation, and GSC maintenance.

Gene knockdown (UAS-RNAi)	WT function	Knockdown location (*nos*-GAL4)	Cytological defect	*Wolbachia* fertility rescue/interaction?	Novel phenotype in this study?
*bgcn*	Bam and Bgcn repress the translation of *nanos*, a gene whose protein is involved in GSC survival and pluripotency through the Nos–Pumilio complex.	GSCs and early CBs	GSC daughter differentiation	Yes	No
*otu*	Bam partners with Otu to form a complex that deubiquitinates Cyclin A (CycA) to promote GSC daughter cell differentiation in females.	GSCs	GSC maintenance	Yes	No
*CycA*	Deubiquitinated to promote GSC daughter cell differentiation in females.	GSCs	GSC daughter differentiation	Yes	No
*Ubi-p63E*	Supplies free ubiquitin and plays an essential role in spermatid differentiation.	GSCs and early CBs	GSC maintenance	Yes	Yes
*CG11700*	Causes partial lethality in females and is predicted to promote ubiquitin protein ligase binding and protein tagging.	GSCs	None observed	No	No
*egg*	Encodes a histone methyltransferase.	GSCs and early CBs	GSC daughter differentiation	Yes	No
*how*	Required for maintenance of CycB (a ubiquitin-related protein), mitosis in GSCs and gonialblasts, and spermatogonial division.	GSCs	GSC maintenance	Yes	Yes
*tut*	Bam binds to Tut and Bgcn proteins to induce autophagy, resulting in spermatogonia differentiation. *Tut* knockdowns using MTD-gal4 result in embryonic lethality.	GSCs and early CBs	GSC maintenance	Yes	Yes
*Rbp9*	Rbp9 inhibits GSC self-renewal and promotes GSC daughter cell proliferation by binding to *bam* mRNA and decreasing Bam protein expression.	GSCs	GSC daughter differentiation	No	No

The candidate gene knockdowns in *bam* WT *W. pipientis*-infected and uninfected flies. The knockdown location (column 3) is dependent on the following Nos-gal4 driver used: a second chromosome GAL4 driver (uninfected of infected with *w*Mel59) that induces the knockdown in GSCs or a third chromosome GAL4 driver (uninfected or infected with *w*MelCS2b) that induces the knockdown in GSCs and GSC cysts.

Next, we assessed the cytological ovarian phenotypes of the remaining candidate gene knockdowns and observed a range in severity of cytological defects. We observed severe cytological germline phenotypes for *Ubi-P63E, otu, egg,* and *tut. Ubi-p63E* and *tut* knockdowns resulted in complete GSC loss in which the entire germline is eventually lost (Table 1, Fig. 7 arrowheads). The ovaries of *egg* knockdown females contained some egg chambers with GSC-like tumors and others with differentiated nurse-cell-like cells but lacked late-stage, mature egg chambers (Table 1, Fig. 7). We observed moderate cytological germline phenotypes for *bgcn, CycA,* and *how* (Fig. 7), consistent with previous findings (McKearin and Spradling 1990; Chen *et al*. 2009; Ni *et al*. 2011; Gao *et al*. 2019). Both *bgcn* and *CycA* knockdowns each featured a mix of properly differentiated egg chambers (Fig. 6 arrows) and egg chambers filled with GSC-like tumors (Fig. 7 arrowheads, Table 1). In *how* knockdown females, we observed some individuals with WT-like ovarian tissue (Fig. 7 arrows), while others contained ovaries with complete germline loss (Table 1, Fig. 7 arrowheads). *Rbp9* knockdown ovaries exhibited very mild cytological defects where most tissue appeared WT (Fig. 7 arrows), but a few egg chambers were a mix of GSC-like tumors and differentiated nurse cells (Fig. 7 arrowhead, Table 1).

Given we confirmed early germline phenotypes for 8 of the 9 candidate gene knockdowns, we next asked whether *W. pipientis* increased the fertility of knockdown females in a similar manner to what we observed for the interaction between *W. pipientis* and the *bam* hypomorph. We measured female fertility in each candidate gene knockdown as the total number of adult progeny per uninfected or *w*Mel59 or *w*MelCS2b *W. pipientis-*infected females (Fig. 7). We found that the independent knockdowns of 4 genes with severe ovarian defects (*Ubi-p63E, otu, egg,* and *tut*) resulted in fertility defects that were rescued by *W. pipientis* (Fig. 7, Table 1). Of the genes with moderate ovarian phenotypes (*bgcn, CycA,* and *how*), infection with *W. pipientis* significantly increased the fertility of *bgcn* and *CycA* knockdown females; however, there was no significant effect of *W. pipientis* infection on the fertility of *how* knockdown females (Fig. 7, Table 1). *W. pipientis* infection did not result in a significant increase in fertility for either *Rbp9* or *CG11700* knockdowns (Fig. 7, Table 1).

## Discussion

Our findings in conjunction with other recent studies (Zong *et al*. 2022; Russell *et al*. 2023) suggest that associations with ubiquitination/deubiquitination are a commonality in the cellular processes with which both *bam* and *W. pipientis* interact. Bam interacts directly with ubiquitin [it is ubiquitinated by Cul2 (Cai *et al*. 2023)]. Bam also binds to its partner Otu to deubiquitinate CycA and promote the differentiation of GSC daughter cells into cystoblasts (Ji *et al*. 2017). While *bgcn* does not have a documented association with ubiquitin, Bgcn expression is necessary for Bam to localize to the fusome, where it is thought to bind Otu and deubiquitinate CycA. Our findings that *bgcn, otu,* and *CycA* are differentially expressed in infected *bam* hypomorph and *bam* null ovaries and that knockdown mutants of all 3 genes in *bam* WT females are rescued by *W. pipientis* infection are evidence that *W. pipientis* manipulates a function common to these genes in GSCs and early differentiating cystoblasts.

While *otu* and *CycA* were also differentially expressed by *W. pipientis* independent of *bam* fertility rescue, we found that these 2 genes were upregulated due to infection in *bam* null ovaries, whereas they were downregulated due to infection in *bam* hypomorphic ovaries (Fig. 5b). Both otu and *CycA* function in GSCs, early cystoblasts, and later in more developed egg chambers. Our *bam* hypomorphic bulk RNA-seq analysis of the entire ovary does not differentiate between these stages. In contrast, *bam* null ovaries do not contain any cystoblasts or egg chambers and thus reflect the effect of *W. pipientis* only in GSC-like cells (Fig. 6a).

Surprisingly, we found that *Ubi-p63E* also plays a role in female GSC maintenance, which has not been previously described (Kashevsky *et al*. 2002; Sugimura and Lilly 2006; Lu *et al*. 2013). *Ubi-p63E* is a polyubiquitin precursor that is known to function in male *D. melanogaster* differentiation and meiosis during spermatogenesis but does not have a reported role in or mutant phenotype in female gametogenesis (Lu *et al*. 2013). As free ubiquitin is needed for CycA turnover, it is possible the *Ubi-p63E* plays a similar role in females as it does in males.

Our observation of a lack of differential expression of *Rbp9* and *tut* in the *bam* null suggests that they may not play a direct role in the fertility rescue by *W. pipientis* in *bam* hypomorph ovaries. *Rbp9* functions downstream of *bam* during GSC daughter cell differentiation (Lu *et al*. 2013; Russell *et al*. 2023). Tut is bound to Bam and Bgcn in males to induce autophagy, which then results in spermatogonia differentiation and, like *Ubi-p63E*, did not have a reported mutant phenotype in female gametogenesis prior to our study (Storto and King 1988; Chen *et al*. 2014). Restoration of *tut* mRNA levels rescued the mutant GSC maintenance phenotype of a *mei-p26* mutant (Russell *et al*. 2023).

Interestingly, genes associated with deubiquitination are also known to trigger cytoplasmic incompatibility in male insects infected with *W. pipientis* (Beckmann *et al*. 2017, 2021). Histone H3K9 trimethylation is known to be involved in the processes of GSC differentiation and maintenance in *D. melanogaster* (Yoon *et al*. 2008; Wang *et al*. 2011; Smolko *et al*. 2018). Three differentially expressed genes specific to the *bam* hypomorph in the histone lysine methylation category (*wde, egg,* and *su(var)3-9*) have known functions in GSC differentiation or maintenance (Yoon *et al*. 2008; Koch *et al*. 2009; Wang *et al*. 2011). *Wde* is a cofactor of *egg* and results in its efficient recruitment to the nucleus and chromatin to perform H3K9 trimethylation (Koch *et al*. 2009). Intriguingly, *egg* is monoubiquitinated in its active methyltransferase form, suggesting a potential interplay between histone lysine methylation and ubiquitination in promoting GSC differentiation or maintenance, both collaboratively and independently (Osumi *et al*. 2019). The activity of the DNA methyltransferase *Dnmt2* has been found to influence pathogen blocking in *D. melanogaster* males, which further emphasizes significance of methylation the fertility rescue by *W. pipientis* in the *bam* hypomorph (Lyko *et al*. 2000; Phalke *et al*. 2009; Bhattacharya *et al*. 2017).

Prior to this study, *W. pipientis* had been reported to interact with the GSC regulating genes *bam*, *Sex-lethal* (*Sxl*), and *meiotic-P26* (*mei-p26*) (Starr and Cline 2002; Flores *et al*. 2015; Bubnell *et al*. 2021; Lindsey *et al*. 2021; Russell *et al*. 2023). *Mei-p26* functions in GSC maintenance, GSC daughter cell differentiation, and meiosis; *W. pipientis* infection rescues the *mei-P26* GSC maintenance mutant phenotype (Li *et al*. 2013; Russell *et al*. 2023). Sxl is necessary for GSC self-renewal and daughter cell differentiation, and *W. pipientis* rescues Sxl GSC daughter cell differentiation defects (Starr and Cline 2002). In Sxl mutants, *W. pipientis* exclusively rescues GSC daughter cell differentiation defects, not the Sxl-induced meiosis defects (Starr and Cline 2002). From our RNAi analysis, 4 of 5 genes that showed a cytological defect (*tut, how, Ubi-p63E,* and *otu*) resulted in GSC-loss mutant phenotypes that were rescued by *W. pipientis*. This underscores the specificity of *W. pipientis* interaction with genes associated with early oogenesis, specifically GSC maintenance and differentiation. We initially hypothesized that *W. pipientis* would primarily rescue *bam's* interaction partners that regulate GSC daughter cell differentiation, as Bam regulates this process. However, as we see that *W. pipientis* additionally rescues GSC-loss phenotypes as shown from our RNAi knockdowns and in a *mei-p26* mutant (Russell *et al*. 2023), our study adds to increasing evidence that *W. pipientis* rescues critical features of GSC maintenance and differentiation through specific host processes (ubiquitination and chromatin modifications).

Because *W. pipientis* is used as a biological control for vector-borne diseases by manipulating reproduction in the host (Iturbe-Ormaetxe *et al*. 2011), it is of particular interest and importance to understand how *W. pipientis* mechanistically interacts with host GSC genes. Using highly inbred isogenic lines of *D. melanogaster* that differ only at a single locus (*bam*) and by *W. pipientis* infection status provided power to identify thousands of differentially expressed genes between infected and uninfected samples across all ages, mating statuses, *bam* genotypes, and *W. pipientis* variants (Supplementary File 1). The functional significance of 8 of the candidates we tested in this study further validates this dataset as an effective resource for understanding the cellular interactions between *W. pipientis* and *Drosophila* oogenesis.

Defining how *W. pipientis* manipulates the germline and reproduction is an essential piece to refining biological controls for infectious disease and crop pests. Additionally, it will be critical to predict the possible functional evolutionary trajectory of new hosts when infected with new variants of *W. pipientis* for this purpose. Understanding the breadth of the cellular interactions between *W. pipientis* and its insect host will be necessary for long-term sustainable use of *W. pipientis* as a biological control tool.

## Supplementary Material

iyae220_Supplementary_Data

## Data Availability

The sequence data generated and used in this study are available in the Sequence Read Archive under BioProject PRJNA1166928 and in the Gene Expression Omnibus under the following accession number GSE280366. Fly lines used in this study are available upon request. The authors affirm that all data necessary for confirming the conclusions of the article are present within the article, figures, and tables. Supplemental material available at GENETICS online.
