# Supplementary material for: Wolbachia pipientis modulates germline stem cells and gene expression associated with ubiquitination and histone lysine trimethylation to rescue fertility defects in Drosophila
Source: Genetics. 2024 Dec 31;229(3):iyae220. doi: 10.1093/genetics/iyae220 (PMC11912866; doi:10.1093/genetics/iyae220)
Supplement: iyae220_Supplementary_Data [file iyae220_supplementary_data.zip › Supplemental_Figure_3_GENETICS-2024-307508.pdf]

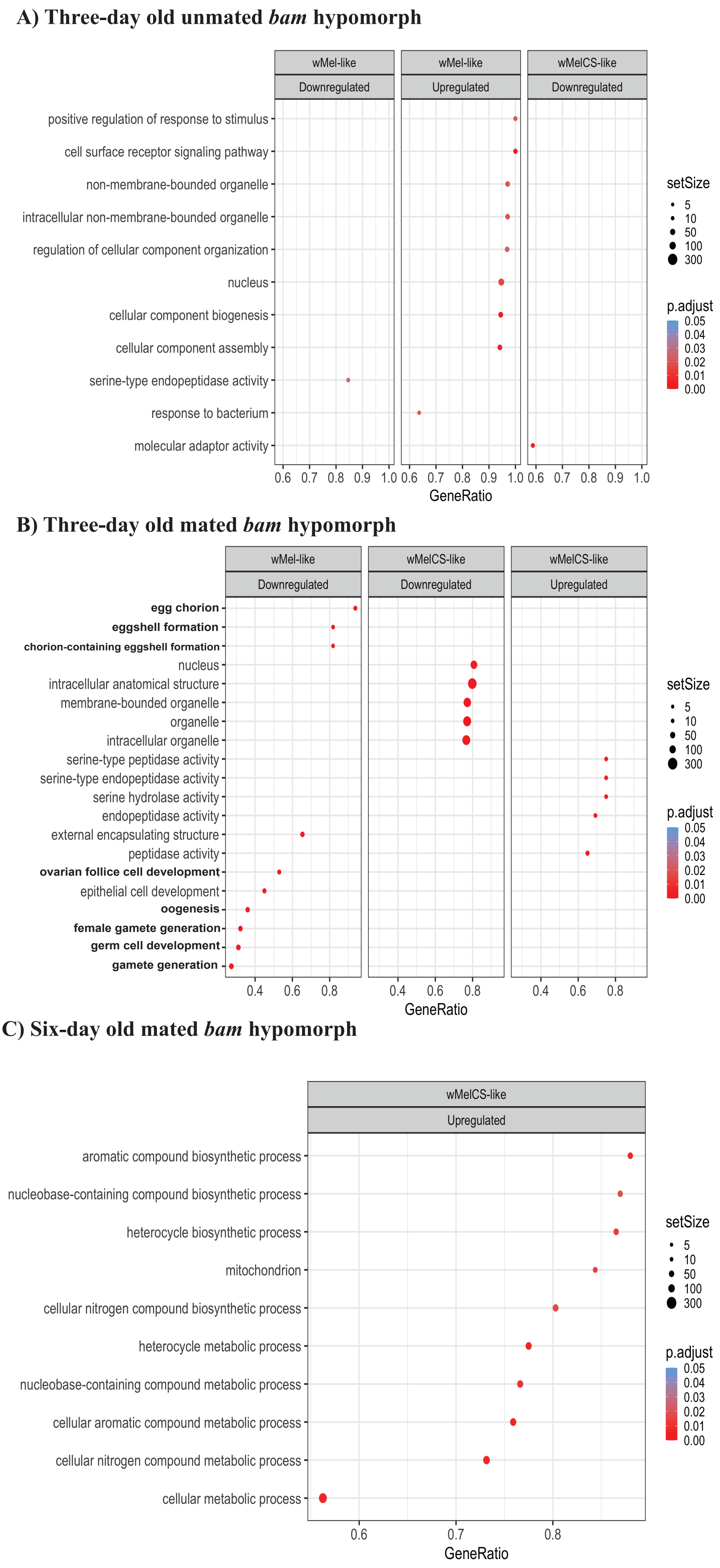
A) Three-day old unmated *bam* hypomorph
wMel-like
Downregulated
wMel-like
Upregulated
wMelCS-like
Downregulated
positive regulation of response to stimulus
cell surface receptor signaling pathway
non-membrane-bounded organelle
intracellular non-membrane-bounded organelle
regulation of cellular component organization
nucleus
cellular component biogenesis
cellular component assembly
serine-type endopeptidase activity
response to bacterium
molecular adaptor activity
0.6 0.7 0.8 0.9 1.0 0.6 0.7 0.8 0.9 1.0 0.6 0.7 0.8 0.9 1.0
GeneRatio
setSize
5
10
50
100
300
p.adjust
0.05
0.04
0.03
0.02
0.01
0.00
B) Three-day old mated *bam* hypomorph
wMel-like
Downregulated
wMelCS-like
Downregulated
wMelCS-like
Upregulated
egg chorion
eggshell formation
chorion-containing eggshell formation
nucleus
intracellular anatomical structure
membrane-bounded organelle
organelle
intracellular organelle
serine-type peptidase activity
serine-type endopeptidase activity
serine hydrolase activity
endopeptidase activity
external encapsulating structure
peptidase activity
ovarian follice cell development
epithelial cell development
oogenesis
female gamete generation
germ cell development
gamete generation
0.4 0.6 0.8 0.4 0.6 0.8 0.4 0.6 0.8
GeneRatio
setSize
5
10
50
100
300
p.adjust
0.05
0.04
0.03
0.02
0.01
0.00
C) Six-day old mated *bam* hypomorph
wMelCS-like
Upregulated
aromatic compound biosynthetic process
nucleobase-containing compound biosynthetic process
heterocycle biosynthetic process
mitochondrion
cellular nitrogen compound biosynthetic process
heterocycle metabolic process
nucleobase-containing compound metabolic process
cellular aromatic compound metabolic process
cellular nitrogen compound metabolic process
cellular metabolic process
0.6
0.7
0.8
GeneRatio
setSize
5
10
50
100
300
p.adjust
0.05
0.04
0.03
0.02
0.01
0.00
