# Supplementary material for: Wolbachia pipientis modulates germline stem cells and gene expression associated with ubiquitination and histone lysine trimethylation to rescue fertility defects in Drosophila
Source: Genetics. 2024 Dec 31;229(3):iyae220. doi: 10.1093/genetics/iyae220 (PMC11912866; doi:10.1093/genetics/iyae220)

A) Unmated three-day old wildtpye *bam*

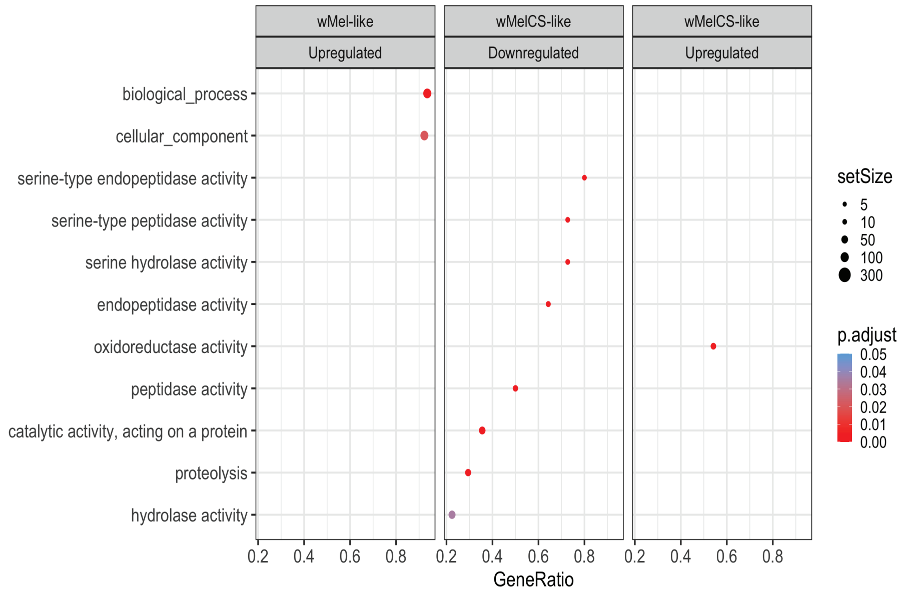

B) Three-day old mated wildtype *bam*

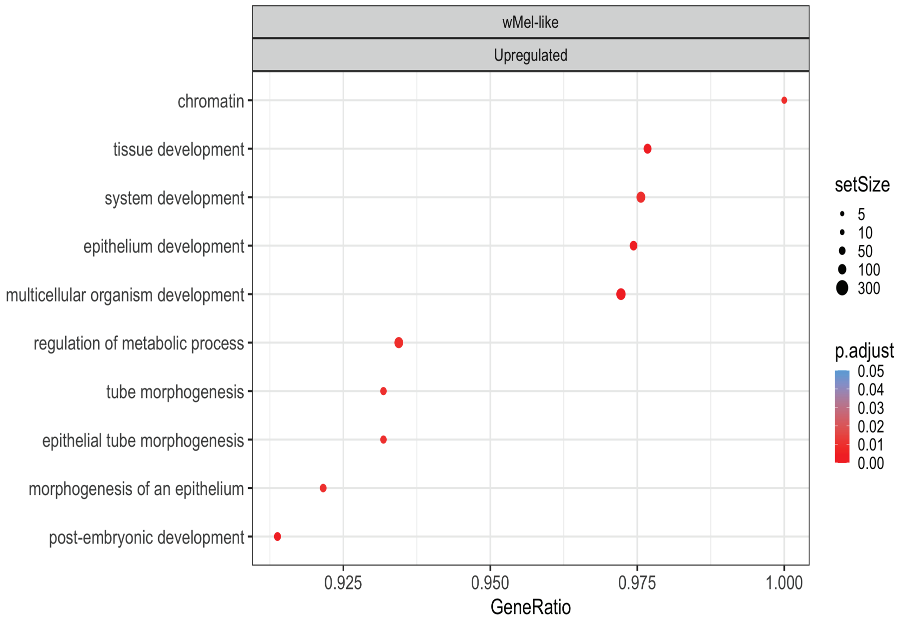

C) Six-day old mated wildtype *bam*

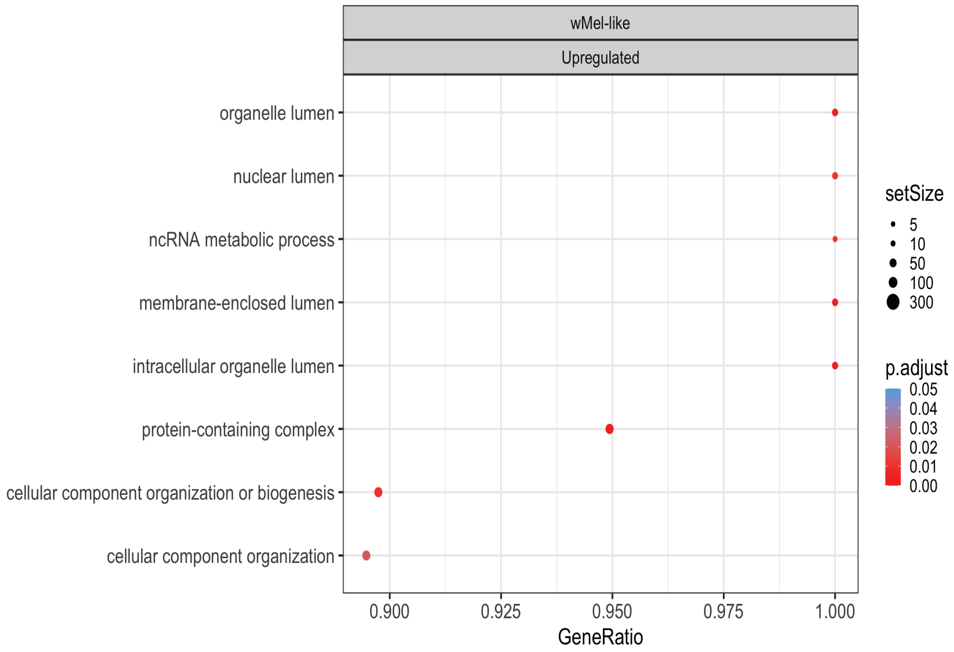

Supplement: iyae220_Supplementary_Data [file iyae220_supplementary_data.zip › Supplemental_Figure_4_GENETICS-2024-307508.pdf]
