# Supplementary material for: Wolbachia pipientis modulates germline stem cells and gene expression associated with ubiquitination and histone lysine trimethylation to rescue fertility defects in Drosophila
Source: Genetics. 2024 Dec 31;229(3):iyae220. doi: 10.1093/genetics/iyae220 (PMC11912866; doi:10.1093/genetics/iyae220)
Supplement: iyae220_Supplementary_Data [file iyae220_supplementary_data.zip › Supplemental_Figure_5_GENETICS-2024-307508.pdf]

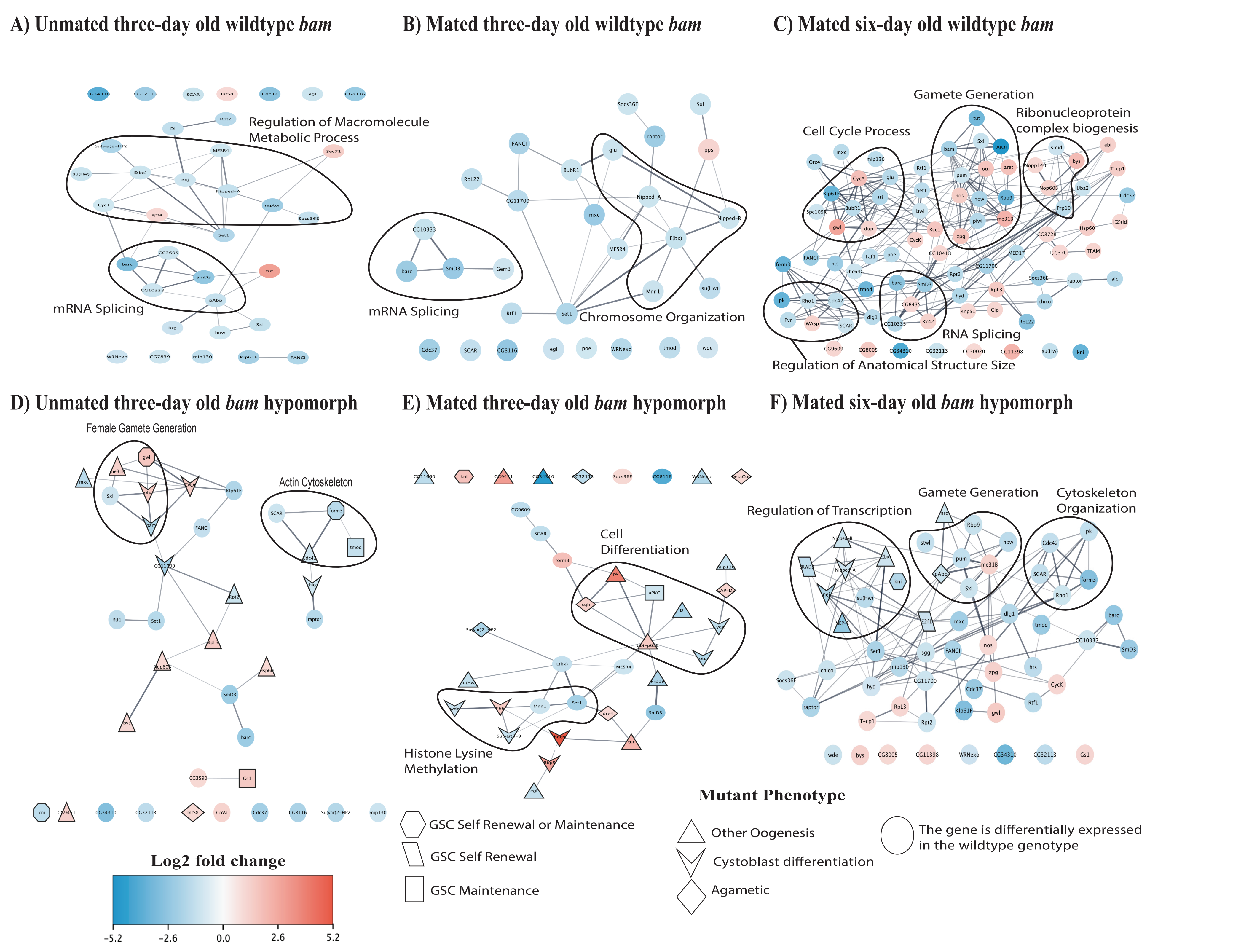
A) Unmated three-day old wildtype *bam*
Regulation of Macromolecule Metabolic Process
mRNA Splicing
B) Mated three-day old wildtype *bam*
mRNA Splicing
Chromosome Organization
C) Mated six-day old wildtype *bam*
Gamete Generation
Ribonucleoprotein complex biogenesis
Cell Cycle Process
RNA Splicing
Regulation of Anatomical Structure Size
D) Unmated three-day old *bam* hypomorph
Female Gamete Generation
Actin Cytoskeleton
E) Mated three-day old *bam* hypomorph
Cell Differentiation
Histone Lysine Methylation
F) Mated six-day old *bam* hypomorph
Regulation of Transcription
Gamete Generation
Cytoskeleton Organization
Log2 fold change
−5.2
−2.6
0.0
2.6
5.2
GSC Self Renewal or Maintenance
GSC Self Renewal
GSC Maintenance
Mutant Phenotype
Other Oogenesis
Cystoblast differentiation
Agametic
The gene is differentially expressed in the wildtype genotype
