# Supplementary material for: Wolbachia pipientis modulates germline stem cells and gene expression associated with ubiquitination and histone lysine trimethylation to rescue fertility defects in Drosophila
Source: Genetics. 2024 Dec 31;229(3):iyae220. doi: 10.1093/genetics/iyae220 (PMC11912866; doi:10.1093/genetics/iyae220)

Supplemental Figure 7

JBrowse Tracks from FlyBase

CG11700 RNA, TRiP RNAi, and reproductive tissue transcriptome data from FlyAtlas2

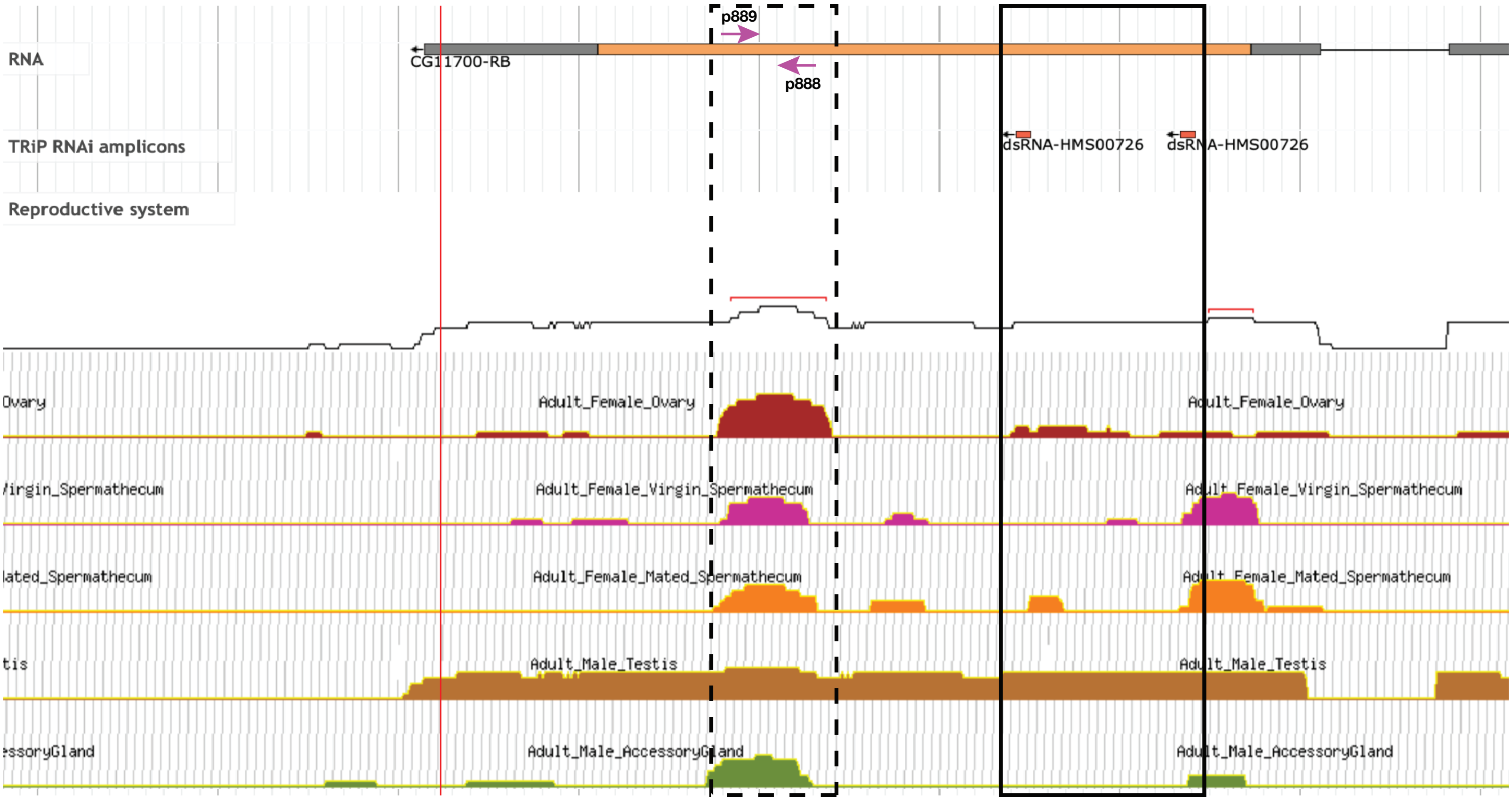

Supplement: iyae220_Supplementary_Data [file iyae220_supplementary_data.zip › Supplemental_Figure_7_GENETICS-2024-307508.pdf]
