# Supplementary material for: Wolbachia pipientis modulates germline stem cells and gene expression associated with ubiquitination and histone lysine trimethylation to rescue fertility defects in Drosophila
Source: Genetics. 2024 Dec 31;229(3):iyae220. doi: 10.1093/genetics/iyae220 (PMC11912866; doi:10.1093/genetics/iyae220)
Supplement: iyae220_Supplementary_Data [file iyae220_supplementary_data.zip › Supplemental_Material_Legends_GENETICS-2024-307508.docx]

**Supplemental Material Legends**

**Supplemental Figure 1. *Bam* wildtype fertility does not correlate with *W. pipientis* titer.**

Wildtype fertility as a response to the combined titer of each *W. pipientis* variant infecting *D. melanogaster*. Fertility and combined *W. pipientis* titer of mated aged flies: (A) three-, (B) six- and (C) nine-day old females. The p-value indicates whether fertility and titer are statistically correlated for each *W. pipientis* type (linear regression model).

**Supplemental Figure 2. A gene network of the 366 GSC genes identified from Yan et al. 2014 and Bam's interactors.**

A modified gene network from Yan et al. (2014) that includes the 366 GSC genes identified from an RNAi screen and 36 of Bam's genetic/physical interaction partners (Cytoscape). Bam's interaction partners that are also included in the 366 GSC genes are highlighted in white. GO categories identified in Yan et al. (2014) are circled.

**Supplemental Figure 3. *W. pipientis* infection-specific *bam* hypomorph gene enrichment (p < 0.05, abs log2 fold change > 1).**

A GO enrichment analysis of genes up or downregulated in each *W. pipientis* group (*w*Mel-like and *w*MelCS-like) infecting the *bam* hypomorph. Initial differential expression analyses were conducted between *bam* hypomorph *D. melanogaster* infected with each *W. pipientis* variant compared to uninfected *D. melanogaster,* but the differentially expressed genes from each *W. pipientis* group were combined for this analysis. A) Unmated three-day old *w*Mel-like and *wMelCS*-like, B) mated three-day old *w*Mel-like and *w*MelCS-like, and C) mated six-day old *w*MelCS-like infected *bam* hypomorph gene enrichment. The set size represents the number of genes within each GO category. The gene ratio is the number of genes within the GO term (set size) divided by the total number of differentially expressed genes. GO categories related to reproduction are highlighted in bold.

**Supplemental Figure 4. *W. pipientis* infection-specific wildtype gene enrichment (p < 0.05).**

A GO enrichment analysis of genes solely in each *W. pipientis* group (*w*Mel-like and *w*MelCS-like) infecting *D. melanogaster*. Initial differential expression analyses were conducted between wildtype *D. melanogaster* infected with each *W. pipientis* variant compared to uninfected *D. melanogaster,* but the differentially expressed genes from each *W. pipientis* group were combined for this analysis. A) Unmated three-day old *w*Mel-like, B) unmated three-day old *w*MelCS-like, C) mated three-day old *w*Mel-like, D) mated three-day old *w*MelCS-like, E) mated six-day old *w*Mel-like, F) and mated six-day old *w*Mel-like infected *D. melanogaster* gene enrichment. The set size represents the number of genes within each GO category. The gene ratio is the number of genes within the GO term (set size) divided by the total number of differentially expressed genes.

**Supplemental Figure 5. Differential expression of GSC genes across all ages/mating status in the wildtype and *bam* hypomorph genotypes.**

Protein-protein networks (Cytoscape) containing the differentially expressed (*D. melanogaster* infected with each *W. pipientis* variant compared to uninfected *D. melanogaster*) GSC genes identified from Yan et al. (2014) in *bam* wildtype A) unmated three-day old, B) mated three-day old, C) mated six-day old flies and in *bam* hypomorph D) unmated three-day old, E) mated three-day old, and F) mated six-day old flies. The average log2 fold change of each differentially expressed gene between flies infected with each *W. pipientis* variant was used because there was not a significant difference between differential expression of these genes in *Drosophila* infected with *w*Mel-like and *w*MelCS-like variants. Genes that are only differentially expressed in the *bam* hypomorph have a black border. The top GO categories that were identified using Cytoscape were circled within each network.

**Supplemental Figure 6. ddCT analysis data and statistics for Figure 5: *W. pipientis* infection results in the differential expression of a subset of RNA-seq candidate genes independent of *bam* rescue.**

Paired delta-delta analysis of candidate genes involved in the rescue of *bam* by *W. pipientis* between infected (*w*MelCS2b) and uninfected *bam* null ovaries using RT-qPCR. The top plot is the raw Ct data for the housekeeping gene (*Rpl32*) and the target gene between each pair (individual sample) with the uninfected samples displayed first on the left and the infected samples displayed next on the right. Sample size is listed below the gene name and reflects three biological replicates with each three technical replicates. The bottom plot shows the effect size of the mean difference for each individual delta and the delta delta with a 95% confidence interval around the mean when comparing infected to uninfected samples for each gene (5000 bootstrap resampling permutation test). The delta-delta Y-axis is inverted negative to positive to highlight that a negative delta-delta CT reflects an increase in transcript abundance.

**Supplemental Figure 7. JBrowse tracks of *CG11700* RNA and reproductive tissue specific expression patterns.** A screenshot from JBrowse (Flybase FB2024_2) illustrating the region of the *CG11700* transcript targeted by the TRiP RNAi line used in this study. Additional tracks show the reproductive tissue specific transcriptome data from FlyAtlas2. The region of *CG11700* targeted by the UAS-RNAi line is not highly expressed in female ovaries (solid line box). The region targeted by our RT-qPCR primers for the data shown in Fig 5 is expressed in female ovaries (dashed line box, arrows with names of primers used in this study (S6 Table).

**Supplemental Figure 8. *W. pipientis*-specific genes included in the gBlock used for absolute quantification of *W. pipientis* titer.**

The *W. pipientis*-specific genes included in our double stranded DNA fragment (gBlock) used for absolute quantification qPCR.

**Supplemental Table 1. Wildtype and *bam* hypomorph fertility assay raw data.**

The number of progeny are listed for the uninfected WT and *bam* hypomorph flies and flies infected with *w*Mel2a, *w*Mel3, *w*MelCS2a, and *w*MelCS2b. The *W. pipientis* variants were grouped into the *w*Mel-like variants (*w*Mel2a and *w*Mel3) and the *w*MelCS-like variants (*w*MelCS2a and *w*MelCS2b) in the “*Wolbachia* type” column. The parents of the progeny were either three-, six- or nine-days old.

**Supplemental Table 2. Genes predicted to be differentially expressed due to ovarian tissue composition, age or mating.**

We computationally predicted what genes could be influenced by ovarian tissue composition, age or mating in mated three- and six-day old WT and *bam* hypomorph flies. Genes that are bolded are Bam’s genetic or physical interactors and genes that are highlighted are GSC genes. Note that the genes listed in this table are not all differentially expressed in our analysis between *W. pipientis* infected and uninfected WT and *bam* hypomorph flies (S2 Table).

**Supplemental Table 3. Downregulation of genes involved in egg formation and oogenesis-related GO categories unique to *bam* hypomorph ovaries infected with *w*Mel-like *W. pipientis* variants.**

The enrichment of differentially expressed genes in the *w*Mel-like (*w*Mel2a and *w*Mel3) infected WT genotypes.

**Supplemental Table 4. Differentially expressed *bam* genetic/physical interactors and GSC genes in the wildtype and *bam* hypomorph genotypes.**

Bam’s differentially expressed genetic and physical interactors and GSC genes in wildtype and *bam* hypomorph unmated three-, mated three-, and mated six-day old flies (p < .05, absolute log2 fold change > 1). Our differential expression analysis compared *W. pipientis* variant infected flies to uninfected flies in the wildtype and *bam* hypomorph genotypes. NA indicates that a given gene was not differentially expressed in each genotype infected with a specific *W. pipientis* variant.

**Supplemental Table 5. Summary of the UAS lines, GAL4 drivers, and final experimental genotypes used in the RNAi knockdowns of the candidate genes.**

Tab 1 includes the UAS genotypes of each candidate gene along with the corresponding BDSC line. Tab 2 contains the GAL4 driver genotypes, their tissue specificity, and their corresponding BDSC line. Tab 3 includes the genotypes of each RNAi knockdown line after crossing the UAS and GAL4 drivers.

**Supplemental Table 6. Primers and gBlock used in this study for *W. pipientis* titer and RT-qPCR.** Tab 1 includes the list of primer names, description, and sequences. Tab 2 includes the name and sequence of the gBlock used to make the plasmid for absolute quantification of *W. pipientis* titer, also see Fig S8.

**Supplemental File 1**

This file contains all of the differentially expressed genes from the RNA-seq analysis in both the wildtype and *bam* hypomorph genotypes. Differential expression was calculated by comparing each *W. pipientis* variant infecting *D. melanogaster* to the uninfected *D. melanogaster* in each genotype. The file contains tabs that separate the differentially expressed genes by the age and mating status of each genotype.
