# Supplementary figures and images for: Wolbachia pipientis modulates germline stem cells and gene expression associated with ubiquitination and histone lysine trimethylation to rescue fertility defects in Drosophila
Source: Genetics. 2024 Dec 31;229(3):iyae220. doi: 10.1093/genetics/iyae220 (PMC11912866; doi:10.1093/genetics/iyae220)

**A) Three-day old wildtype *bam***

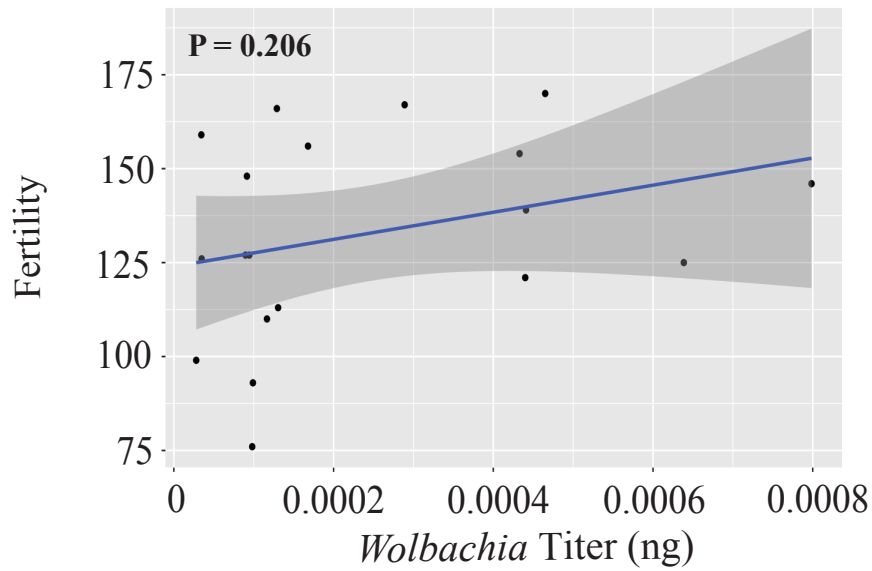

**B) Six-day old wildtype *bam***

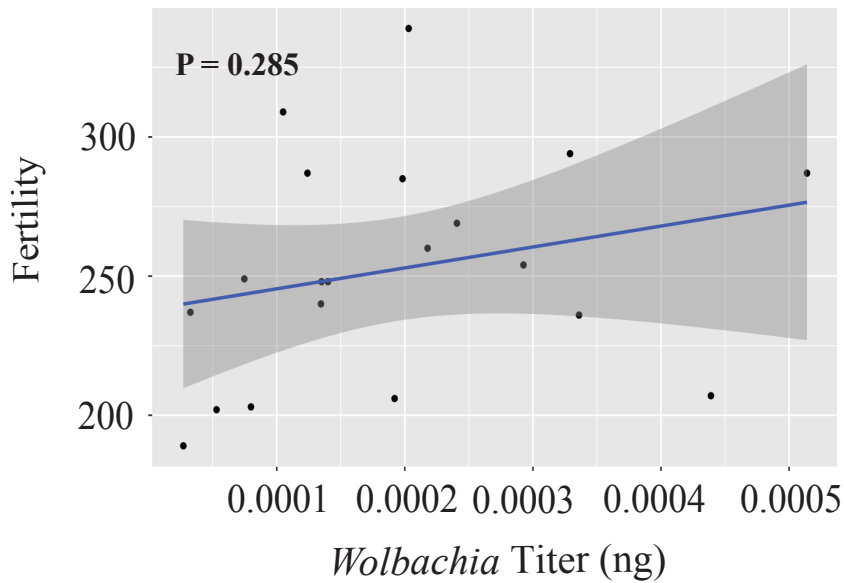

**C) Nine-day old wildtype *bam***

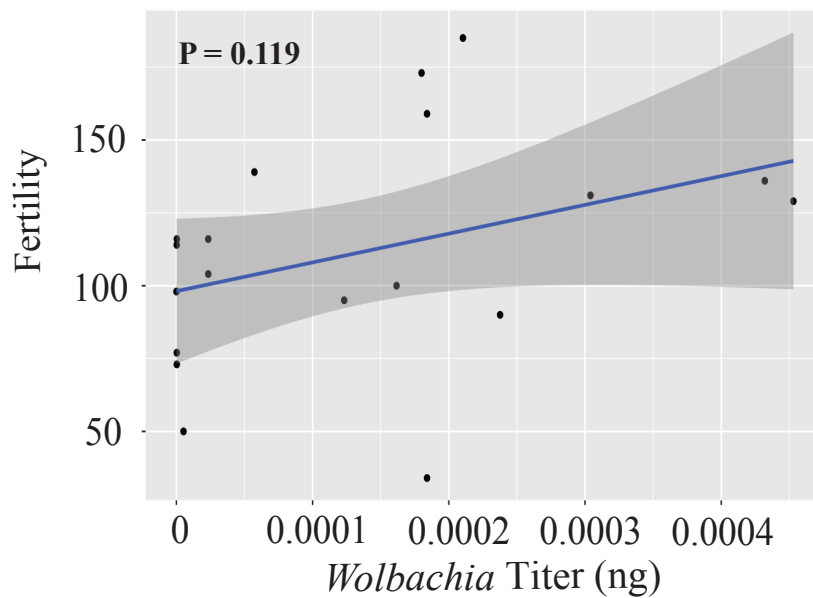

Supplement: iyae220_Supplementary_Data [file iyae220_supplementary_data.zip › Supplemental_Figure_1_GENETICS-2024-307508.pdf]

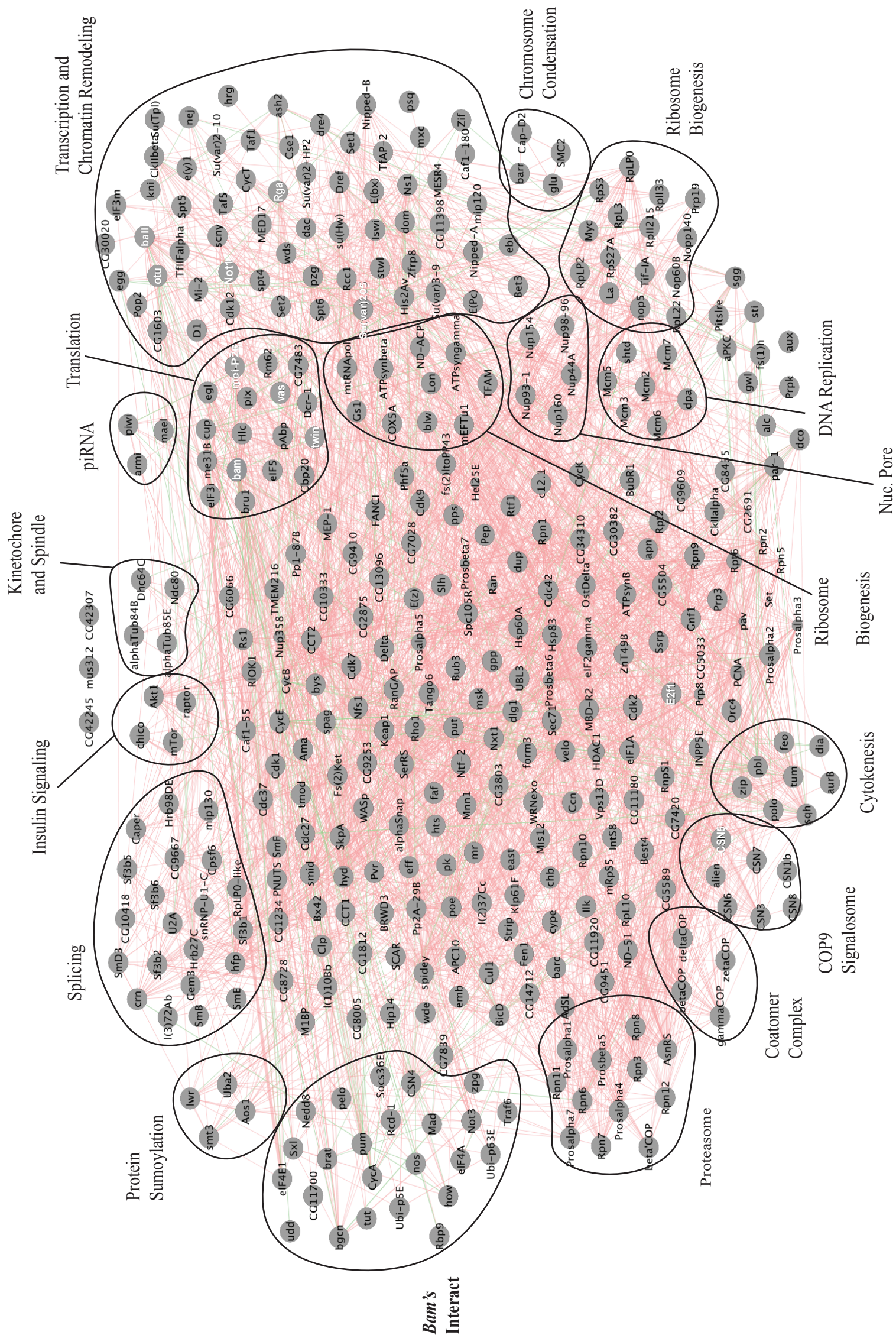

Supplement: iyae220_Supplementary_Data [file iyae220_supplementary_data.zip › Supplemental_Figure_2_GENETICS-2024-307508.pdf]

deltadelta analysis for RTqPCR of candidate genes in *bam* null ovaries

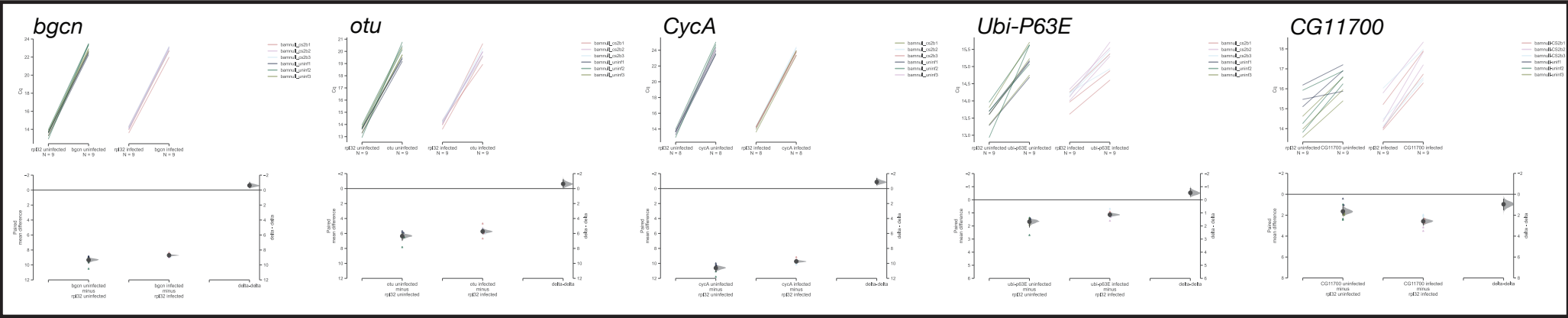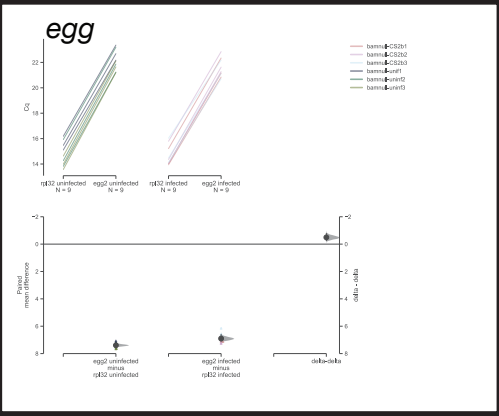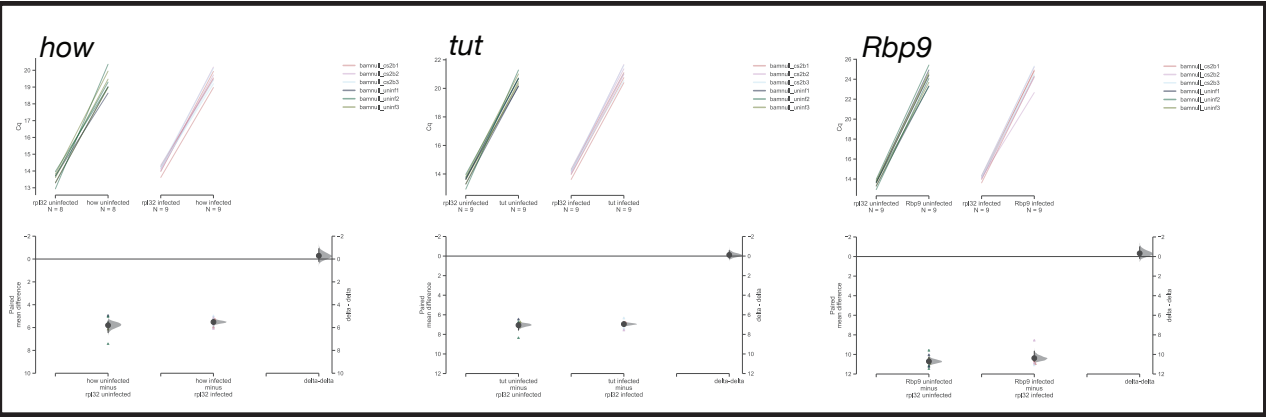

Supplement: iyae220_Supplementary_Data [file iyae220_supplementary_data.zip › Supplemental_Figure_6_GENETICS-2024-307508.pdf]

Supplemental Figure 8

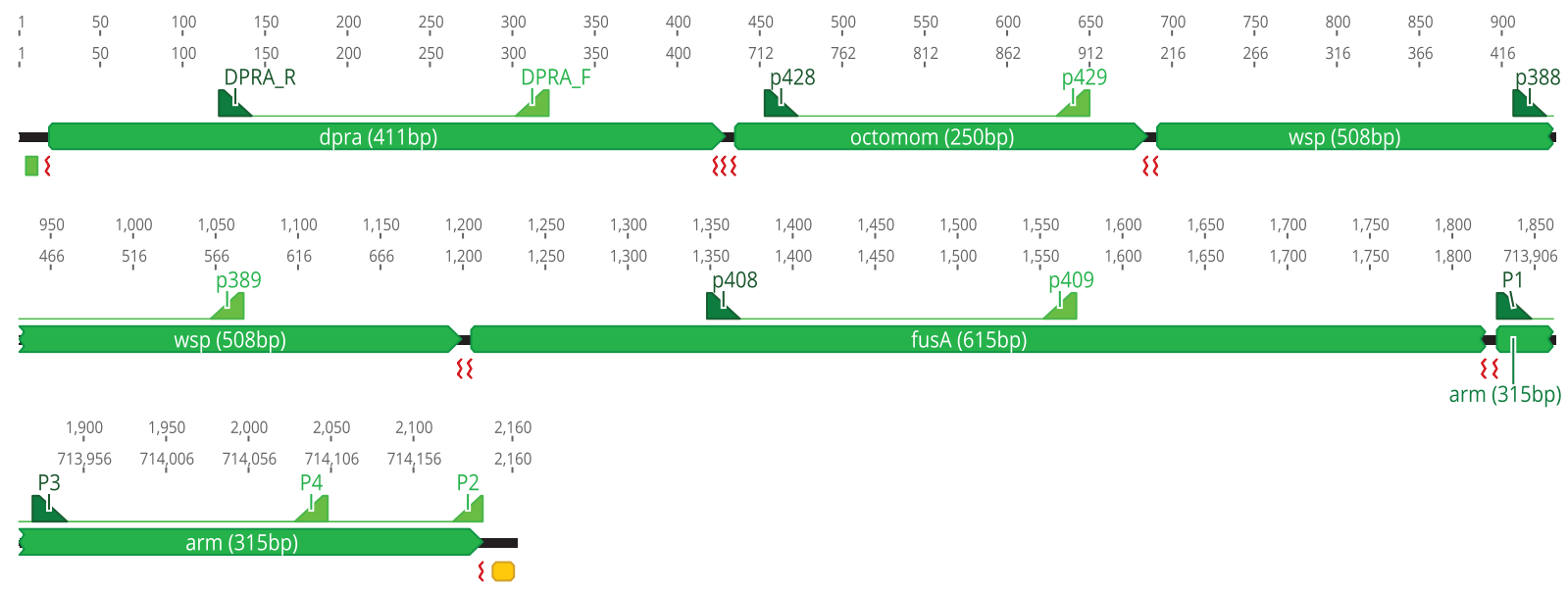

Supplement: iyae220_Supplementary_Data [file iyae220_supplementary_data.zip › Supplemental_Figure_8_GENETICS-2024-307508.pdf]
